# The GFI1–FOXO1 axis regulates NK cell maturation and function

**DOI:** 10.1038/s41467-026-72022-6

**Published:** 2026-04-22

**Authors:** Qiutong Huang, M. Zeeshan Chaudhry, James Dight, Louisa Alim, Huiyang Yu, Renae Denman, Hiu On Man, Jaring Schreuder, Alexandra L. Garnham, Zewen K. Tuong, Fernando Souza-Fonseca-Guimaraes, Nicolas Jacquelot, Gabrielle T. Belz

**Affiliations:** 1 The University of Queensland Frazer Institute, University of Queensland, Woolloongabba, Queensland Australia; 2 Walter and Eliza Hall Institute of Medical Research, 1G Royal Parade, Parkville, Victoria Australia; 3 Department of Medical Biology, University of Melbourne, Victoria, Australia; 4 Riddell Centre for Cancer Immunotherapy, Arnie Charbonneau Cancer Institute, University of Calgary, Calgary, Alberta Canada; 5 Ian Frazer Centre for Children’s Immunotherapy Research, Child Health Research Centre, The University of Queensland, South Brisbane, Queensland Australia

**Keywords:** Epigenetics in immune cells, NK cells

## Abstract

Natural killer cells defend against malignancies and viral infections through a tightly controlled program of differentiation and maturation. However, the transcriptional mechanisms guiding this process remain incompletely defined. Using paired single-cell multiomic profiling, we identify GFI1 as an epigenetic regulator of NK cell differentiation, coordinating EOMES and T-BET transcriptional balance to promote NK cell proliferation and the transition from immature to terminally differentiated NK cell states. GFI1 represses FOXO1 chromatin accessibility in mature NK cells, which normally limits NK cell proliferation and maturation. Co-deletion of both GFI1 and FOXO1 largely rescues NK cell differentiation, identifying a critical GFI1–FOXO1 axis required for protection against tumour metastasis. These findings position GFI1 as a key transcriptional node integrating NK cell differentiation, activation and effector programs.

## Abstract (web summary)

NK cell differentiation is critical for immune defence but its transcriptional control remains unclear. The authors here define a GFI1–FOXO1 axis that balances EOMES/T-BET to drive NK cell maturation, proliferation and tumour control.

## Introduction

Natural killer (NK) cells are critical for immune surveillance against tumours and viral infections. Their capacity to rapidly sense cellular stress and initiate cytotoxic and cytokine responses limits pathogen spread and restrains tumour progression. These protective functions depend on the generation of mature peripheral NK cells equipped with a repertoire of effector mechanisms^1,2^.

Murine NK cells arise from the common lymphoid progenitor and progress through NK precursor and immature stages in the bone marrow before differentiating into mature NK subsets in peripheral tissues. The process is intrinsically guided by stage-specific transcriptional regulators that control (i) early development (e.g., *Spi1*^3^ and *Ets1*^4^), (ii) the immature to mature NK transition (e.g., *Id2*^5^ and *Tcf7*^6^), and (iii) terminal maturation (e.g., *Foxo1*^7^, *Zeb2*^8^*, Eomes* and *Tbx21*). Terminal differentiation proceeds through discrete immature (Imm), M1 and M2 developmental stages defined by differential expression of the integrin CD11b (*Itgam*), CD27 and KLRG1^9–11^. Although these transcription factors promote NK cell maturation and effector development, mechanisms that also restrain excessive cytokine and cytolytic activity are essential to calibrate NK cell responses and prevent immunopathology^7,8,12^.

How different transcriptional cues are integrated to coordinate NK cell maturation and functional competence remains incompletely understood. T-BET (*Tbx21*) promotes late NK cell maturation and effector function^13^, while FOXO1 acts upstream as a ‘brake’ on the differentiation programme^7^. However, the circuitry that links these pathways is unclear. Growth factor independent-1 (GFI1) is a transcriptional repressor expressed in early bone marrow progenitors^14,15^ where it directs cell fate decisions in myeloid and B cell compartments and is required for the generation or maintenance of multiple lymphoid and myeloid populations, including group 2 innate lymphoid cells^16^, dendritic cells^17^ and memory CD8^+^ T cells^18,19^. Despite evidence for GFI1 expression in progenitor cells^20^, its role in NK cell differentiation or function has not been defined.

Here, we identify GFI1 as an intrinsic regulator of NK cell differentiation. We show that GFI1 expression varies across NK cell subsets and is required for the generation of mature M1 (CD11b⁺KLRG1⁻) and M2 (CD11b⁺KLRG1⁺) NK cells, where it coordinates transcriptional and chromatin programmes controlling proliferation and terminal maturation. Mechanistically, GFI1 balances EOMES, FOXO1 and T-BET to regulate NK cell developmental progression and loss of FOXO1 substantially rescues differentiation defects caused by GFI1 deficiency, revealing a GFI1-FOXO1 regulatory axis. These findings identify a transcriptional circuit that integrates opposing regulatory inputs to control NK cell maturation and function. More broadly, this work provides insight to how transcriptional networks shape innate lymphocyte differentiation and may inform strategies to enhance NK-cell mediated anti-tumour immunity.

## Results

### Intrinsic GFI1 controls NK cell development and differentiation

**Fig. 1:**
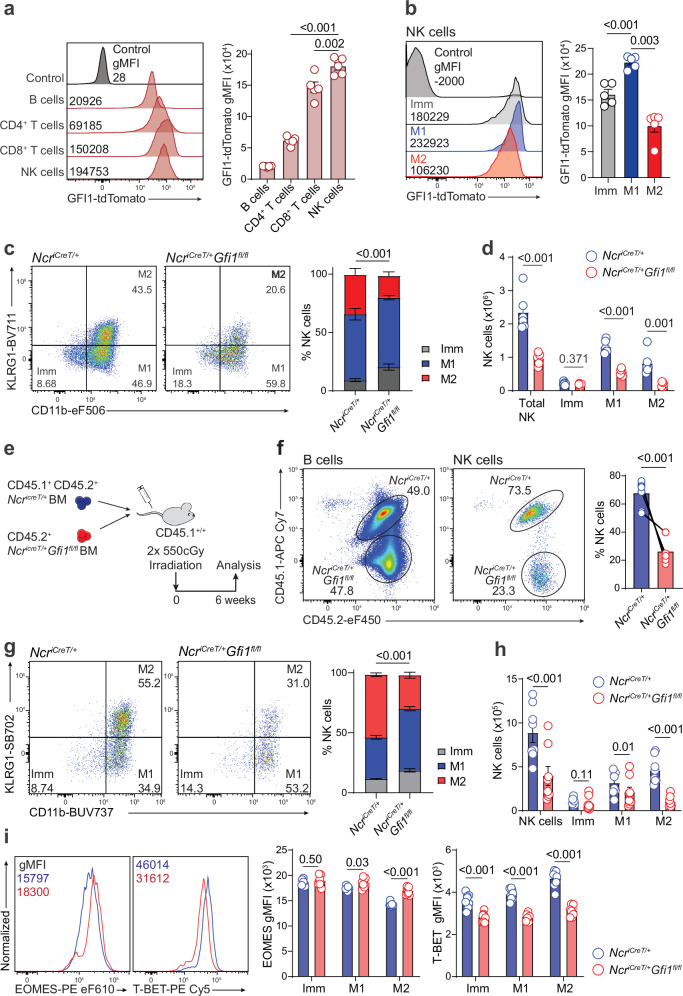
Intrinsic GFI1 expression is required for NK cell differentiation. **a** Histograms (left) and geometric mean fluorescence intensity (gMFI) quantification (right) of GFI1-tdTomato expression in B cells (live CD3^-^CD19^+^), CD4^+^ T cells (live CD19^-^CD3^+^CD4^+^CD8a^-^), CD8^+^ T cells (live CD19^-^CD3^+^CD4^-^CD8a^+^) and NK cells (live CD3^-^TCRβ^-^CD19^-^NK1.1^+^NKp46^+^CD49b^+^) isolated from the spleen of naive *Gfi1*^*tdTomato*/+^ mice. Control NK cells were isolated from the spleen of naïve C57BL/6 mice. **b** Histograms (left) and gMFI quantification (right) of GFI1-tdTomato expression in immature (Imm, CD11b^-^KLRG1^-^), M1 (CD11b^+^KLRG1^-^) and M2 (CD11b^+^KLRG1^+^) NK cells isolated from the spleen of naive *Gfi1*^*tdTomato*/+^ mice. Control NK cells were isolated from the spleen of naïve C57BL/6 mice. **a**,** b** Data show mean ± s.e.m. for one of two independent experiments (*n* = 5 individual mice/experiment). **c** Flow cytometric dot plots (left) and bar plots (right) show frequency of NK cell subsets among total splenic NK cells isolated from naïve *Ncr*^*iCreT/+*^ and *Ncr*^*iCreT/+*^*Gfi1*^*fl/fl*^ mice. **d** Total number of splenic NK cell subsets isolated from naïve *Ncr*^*iCreT/+*^ and *Ncr*^*iCreT/+*^*Gfi1*^*fl/fl*^ mice. **c**, **d** Data from one of five independent experiments shown as mean ± s.e.m. (*n* = 6 individual mice/experiment). **e** Schematic of bone marrow chimeric mice generation. **f** Flow cytometric dot plots show frequency of donor *Ncr*^*iCreT/+*^ (CD45.1^+^CD45.2^+^) and *Ncr*^*iCreT/+*^*Gfi1*^*fl/fl*^ (CD45.2^+^) splenic B and NK cells 6 weeks after bone marrow reconstitution (left). Frequency of NK cells isolated from the spleen of naïve bone marrow chimeric mice (right), as in (**e**). **g** Representative plot (right) and quantification (left) of donor NK cell subsets frequency in the spleen as in (**f**). **h** Total number of splenic donor NK cell subsets as in (**f**). **i** Histograms (left) and gMFI quantification (right) of EOMES and T-BET expression in donor splenic NK cells as in (**f**). **f**–**i** Data pooled from two independent experiments shown as mean ± s.e.m. (*n* = 8 mice). Statistical significance was determined using a one-way ANOVA and Tukey’s post-test (**a**, **b**), a two-tailed Student’s *t* test (**c**, **d**), or a two-tailed paired *t* test (**f**–**i**).

To understand how GFI1 is regulated in NK cells, we quantitated tdTomato reporter expression in naïve *Gfi1*^*tdTomato/+*^ mice in which tdTomato is expressed under control of the endogenous GFI1 promoter^21^ (Supplementary Fig. 1a). NK cells expressed higher levels of GFI1-tdTomato expression than CD4^+^ and CD8^+^ T cells (Fig. 1a). Bone marrow (BM) NK cells expressed lower levels of GFI1-tdTomato than spleen, liver and lung NK cells (Supplementary Fig. 1b) and GFI1-tdTomato expression in NK cells was inversely correlated with the frequency of immature (Imm, CD11b^-^KLRG1^-^) NK cells (Supplementary Fig. 1c) suggesting that GFI1 expression increases with NK cell maturation. To ascertain this, GFI1-tdTomato expression was analysed in splenic NK cell subsets, revealing that CD11b^+^KLRG1^-^ M1 cells expressed significantly higher GFI1-tdTomato than Imm and CD11b^+^KLRG1^+^ M2 NK cells (Fig. 1b). This is consistent with a potential checkpoint at the Imm to M1 transition. NK cells isolated from the liver and lungs also showed significantly higher GFI1-tdTomato expression in M1 NK cells, but not in BM NK cell subsets (Supplementary Fig. 1d). Thus, GFI1 expression correlates with NK cell differentiation and maturation in peripheral tissues.

Next, we tested whether GFI1 was required for NK cell development and differentiation. At steady-state, NK cell-specific deletion (using *Ncr1*^*tm1.1(icre)Viv*^ referred to as *Ncr*^*iCreT/+*^) of GFI1^22^ (*Ncr*^*iCreT/+*^*Gfi1*^*fl/fl*^ mice) showed an increase in the frequency of Imm NK cells and reduced frequency of M2 cells in the spleen compared with controls (*Ncr*^*iCreT/+*^ mice)(Fig. 1c). The number of M1 and M2 NK cells were reduced in the absence of GFI1 in spleen, lungs and liver (Fig. 1d and Supplementary Fig. 1e). These data indicate that loss of GFI1 in NK cells impaired differentiation of Imm NK cells into M1 and M2 NK cells.

To determine whether this requirement was cell intrinsic, we generated mixed bone marrow chimeras using congenically-marked *Ncr*^*iCreT/+*^ (CD45.1^+^CD45.2^+^) and *Ncr*^*iCreT/+*^*Gfi1*^*fl/fl*^ (CD45.2^+^) donor BM combined in an equal ratio (1:1) which was then transplanted into lethally-irradiated CD45.1^+^ mice (Fig. 1e). Reconstitution of chimeras resulted in the development of B cells equally from each donor BM, while the frequency of NK cells developing from *Ncr*^*iCreT/+*^*Gfi1*^*fl/fl*^ BM was significantly reduced compared with wildtype bone marrow (*Ncr*^*iCreT/+*^) (Fig. 1f). This was accompanied by both a lower frequency (Fig. 1g) and total number of mature NK cells (Fig. 1h). This reduction was most marked in the M2 NK subset across spleen, lungs and liver (Fig. 1h and Supplementary Fig. 1f). In contrast to spleen and lungs, total Imm NK cells were reduced in BM by a lack of GFI1 (Supplementary Fig. 1f). Consistent with altered differentiation, GFI1-deficient NK cells displayed increased EOMES and reduced T-BET across all NK cell subsets compared with wildtype (*Ncr*^*iCreT/+*^) NK cells (Fig. 1i). Together, these data establish a cell-intrinsic requirement for GFI1 in NK cell differentiation and transcriptional programming.

### GFI1 programmes NK cell differentiation

**Fig. 2:**
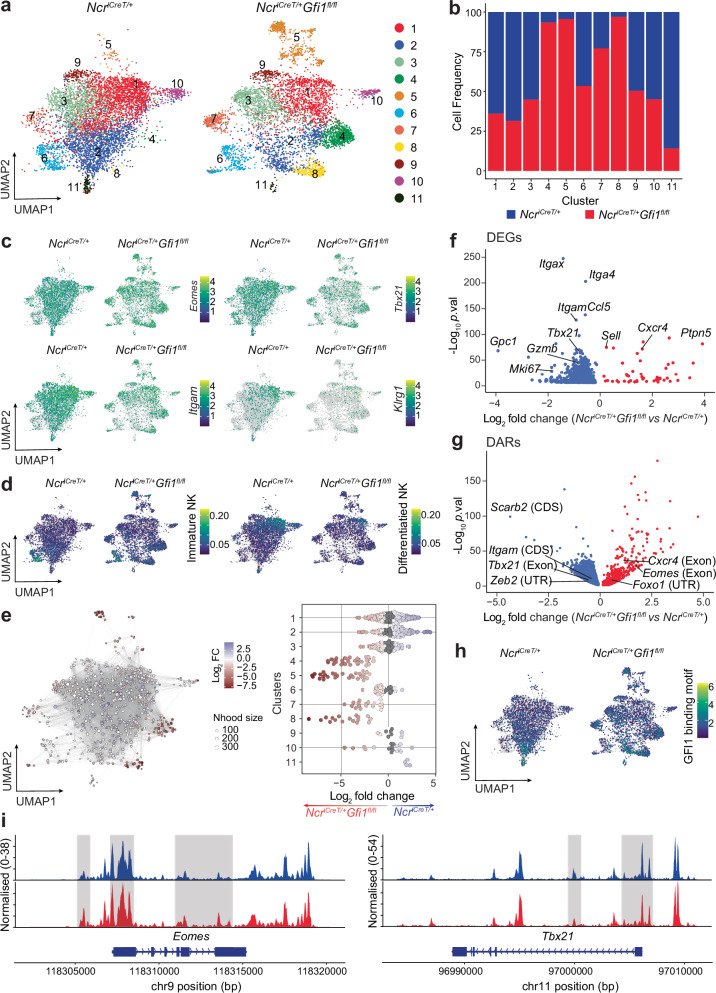
scMultiomic-seq delineated GFI1-mediated epigenetic and transcriptional regulation of NK cell differentiation. **a** UMAP shows scRNA-seq based unsupervised clustering of splenic *Ncr*^*iCreT/+*^ (*n* = 6225 cells) and *Ncr*^*iCreT/+*^*Gfi1*^*fl/fl*^ (*n* = 5654 cells) NK cells isolated from naïve mice. **b** Frequency of *Ncr*^*iCreT/+*^ and *Ncr*^*iCreT/+*^*Gfi1*^*fl/fl*^ NK cells in each cluster identified in panel (**a**). **c** UMAP showing normalised RNA expression of *Eomes, Tbx21, Itgam* and *Klrg1* in NK cells as in (**a**). **d** UMAP showing immature and differentiated NK cell gene expression signature enrichment as in (**a**). **e** Differential neighbourhood abundance (Log_2_FC), calculated using scRNA-seq data, is shown on the UMAP (right) and beeswarm plots (left). Blue and red dots indicate neighbourhoods significant enriched (*p* < 0.05, negative binomial generalised linear model testing with spatial FDR correction) for *Ncr*^*iCreT/+*^ and *Ncr*^*iCreT/+*^*Gfi1*^*fl/fl*^ NK cells, respectively, while grey dots represent neighbourhoods without significant enrichment (*p* > 0.05). **f**–**g** Differentially expressed genes (**f**) and differentially accessible regions (**g**) in total NK cells detected using scRNA-seq and scATAC-seq pseudobulk analysis, respectively. Blue dots indicate genes (DEGs) or regions (DARs) significantly (*p* < 0.05, Wilcoxon rank-sum test with Bonferroni post-test) upregulated in *Ncr*^*iCreT/+*^ NK cells, while red dots indicate those significantly upregulated in *Ncr*^*iCreT/+*^*Gfi1*^*fl/fl*^ NK cells. **h** GFI1 DNA binding motif enrichment in chromatin peaks identified using scATAC-seq as in (**a**). **i** Cluster 1 *Ncr*^*iCreT/+*^ (blue histogram) and *Ncr*^*iCreT/+*^*Gfi1*^*fl/fl*^ (red histogram) NK cell chromatin accessibility at *Eomes* (left) and *Tbx21* (right) gene loci as in (**a**). Grey boxes highlight differentially accessible chromatin regions.

To understand the NK cell molecular programmes regulated by GFI1, splenic NK cells were purified from naïve *Ncr*^*iCreT/+*^ and *Ncr*^*iCreT/+*^*Gfi1*^*fl/fl*^ mice, and single-cell multiomic sequencing (scMultiomic-seq), which combines paired transcriptional and epigenetic analysis, was performed. Uniform manifold approximation and projection (UMAP) and unsupervised clustering of reciprocal principal component analysis (RPCA) integrated wildtype (*Ncr*^*iCreT/+*^) and GFI1-deficient NK cells resolved them into 11 clusters based on their transcriptional profile (Fig. 2a). Cluster 1, 2 and 11 were enriched with *Ncr*^*iCreT/+*^cells, cluster 4, 5, 7 and 8 had higher number of GFI1-deficient NK cells, while cluster 3, 6, 9 and 10 had comparable numbers of both genotypes (Fig. 2b). Cluster 1 and 10 showed high levels of *Itgam* (CD11b), *Klrg1* and *Zeb2* expression (Fig. 2c and Supplementary Fig. 2a), identifying them as terminally differentiated M2-like NK cell population. Cluster 10 further displayed elevated levels of *Itgax* (CD11c) and *Itga2* (CD49b), predominantly enriched in *Ncr*^*iCreT/+*^ NK cells. Cluster 6 exhibited the highest expression of *Bcl11b* and *Il7r*, while *Itgam*, *Gzma* and *Zeb2* expression was the lowest (Supplementary Fig. 2a, b), consistent with an immature NK cell cluster. Cluster 2, 3 and 9 had higher levels of *Itgam*, but relatively lower levels of *Klrg1*, consistent with the intermediate (M1-like) stage of NK cell differentiation. To further assess the differentiation status of NK cell clusters, we applied CD11b^+^KLRG1^-^ immature and CD11b^+^KLRG1^+^ differentiated NK cell signature to our single-cell clusters. This showed that cluster 1 and 10 cells had terminally differentiated NK cell signature, whereas cluster 6 cells were enriched for immature NK cell signature (Fig. 2d). Cluster 4, 5, 7 and 8 that are enriched for *Ncr*^*iCreT/+*^*Gfi1*^*fl/fl*^ cells (Fig. 2b), exhibited a transcriptional signature similar to immature NK cells (Fig. 2d), with elevated *Eomes* expression (Fig. 2c). Comparing differential abundance of cellular neighbourhoods using Milo^23^ revealed that neighbourhoods in immature NK cells (cluster 6) were significantly enriched for GFI1-deficient NK cells (Fig. 2e), although the frequency of both genotype cells was similar in this cluster (Fig. 2b). In contrast, local enrichment of *Ncr*^*iCreT/+*^ NK cells was detected in cluster 2 and 11. Notably, cluster 11 was identified as proliferating cells as it had high expression of cell cycle-related genes, such as *Mki67*, *Top2a* and E2F family members (Supplementary Fig. 2a), but this expression pattern was restricted to *Ncr*^*iCreT/+*^ cells. These data suggest that GFI1 drives the NK differentiation programme, as GFI1-deficient NK cells were largely restricted to an immature NK cell transcriptional profile.

Pseudobulk scRNA-seq analysis showed reduced expression of key transcription factors involved in NK cell differentiation, such as *Tbx21* and *Zeb2*, in GFI1-deficient cells (Fig. 2f and Supplementary Fig. 2a). The expression of these genes remained reduced even in mature *Ncr*^*iCreT/+*^*Gfi1*^*fl/fl*^ NK cells within cluster 1 (Supplementary Fig. 2a, b), while expression of *Foxo1*, a known suppressor of NK cell differentiation^7^, was elevated in *Ncr*^*iCreT/+*^*Gfi1*^*fl/fl*^ NK cell enriched clusters (Supplementary Fig. 2b). Moreover, *Itgam*, *Klrg1*, *Mki67*, *Gzmb* and *Ifng* expression was reduced in GFI1-deficient NK cells globally as well as cluster 1 and 6 cells (Supplementary Data 1), suggesting that GFI1 promotes NK cell proliferation, cytotoxicity and differentiation. These findings were further supported by bulk RNA-seq analysis of *Ncr*^*iCreT/+*^ and *Ncr*^*iCreT/+*^*Gfi1*^*fl/fl*^ NK cell subsets showing reduced *Tbx21* and cell cycle-related gene expression in differentiated *Ncr*^*iCreT/+*^*Gfi1*^*fl/fl*^ NK cells together with elevated expression of *Eomes* and *Foxo1* in all *Ncr*^*iCreT/+*^*Gfi1*^*fl/fl*^ NK cell subsets (Supplementary Fig. 2c and Supplementary Data 2). Moreover, loss of functional GFI1 led to increased *Gfi1* transcripts, confirming an autoregulatory feedback loop^24^. These transcriptional changes were associated with an altered chromatin accessibility profile in GFI1-deficeint cells (Fig. 2g and Supplementary Data 3). Chromatin accessibility at key genetic loci associated with NK cell maturation, such as *Tbx21*, *Zeb2*, *Klrg1* and *Itgam,* was reduced in GFI1-deficient NK cells (Fig. 2g and Supplementary Fig. 2d), while *Eomes* and *Foxo1* loci displayed increased epigenetic accessibility. Motif enrichment analysis further demonstrated the epigenetic changes, with significant enrichment of GFI1 binding motif within clusters dominated by GFI1-deficent NK cells (Fig. 2h), suggesting that the altered gene expression in these clusters is linked to a lack of GFI1 binding motif engagement. In addition to global changes in epigenetic profile, mature NK cells (cluster 1) lacking GFI1 displayed dysregulated accessibility at key NK differentiation regulator gene loci, with increased accessibility at the *Eomes* locus and reduced accessibility at *Tbx21* (Fig. 2i and Supplementary Fig. 3). Together, these data show that GFI1 regulates the NK cell chromatin landscape and promotes NK cell differentiation by regulating crucial NK cell genes such as *Eomes* and *Tbx21*.

### Loss of GFI1 impairs NK cell proliferation and surface receptor expression

**Fig. 3:**
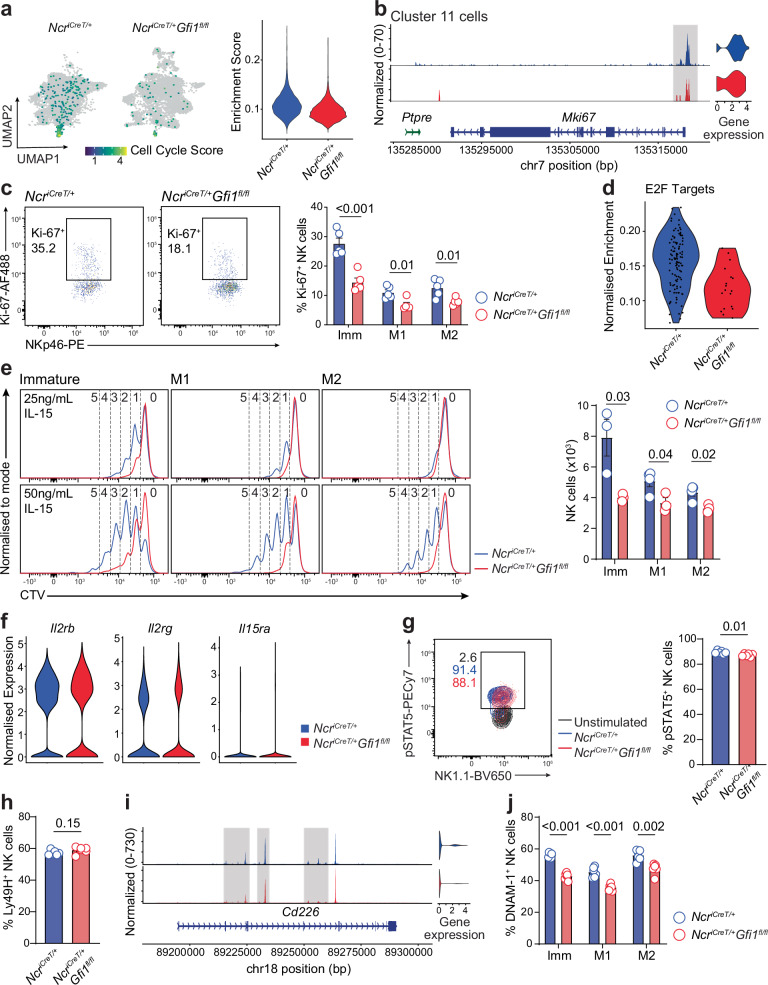
GFI1-deficient NK cells exhibit reduced proliferation and aberrant NK cell activatory receptor expression. **a** UMAP (left) and violin plots (right) showing KEGG cell cycle pathway enrichment score in splenic NK cells isolated from naïve *Ncr*^*iCreT/+*^ and *Ncr*^*iCreT/+*^*Gfi1*^*fl/fl*^ mice for scMultiomic-seq. **b** Cluster 11 *Ncr*^*iCreT/+*^ (blue histogram and violin plots) and *Ncr*^*iCreT/+*^*Gfi1*^*fl/fl*^ (red histogram and violin plots) NK cell chromatin accessibility and gene expression at the *Mki67* gene locus as in (**a**). Grey box highlights a differentially accessible chromatin region. **c** Representative plots (left) and quantification (right) showing frequency of Ki-67^+^ cells among total NK cells isolated from the spleen of naïve mice. Data show the mean ± s.e.m. pooled from two independent experiments (*n* = 5 mice). **d** Hallmark E2F target gene enrichment score in cluster 11 NK cells as in (**a**). **e** Proliferation of CTV-labelled NK cell subsets purified from the spleen of naïve mice cultured in vitro with 25 ng/mL or 50 ng/mL of recombinant IL-15 for 5 days. Dotted lines mark cell divisions (right panel). Enumeration of NK cells after 5 days of in vitro culture (right panel). Data show mean cells/well ± s.e.m. for one of two independent experiments (*n* = 3 mice/experiment). **f** Normalised gene expression in splenic NK cells as in (**a**). **g** Flow cytometric plot (left) and quantification (right) show frequency of phosphorylated STAT5 (pSTAT5) in splenic NK cells following 30 min in vitro culture with recombinant 50 ng/mL IL-15 as in (**e**). Data shown as mean ± s.e.m. for one of two independent experiments (*n* = 6 mice/experiment). **h** Frequency of splenic Ly49H^+^ NK cells isolated from naïve mice. Data shown as mean ± s.e.m. for one of three independent experiments (*n* = 6 mice). **i** Total *Ncr*^*iCreT/+*^ (blue histogram and violin plots) and *Ncr*^*iCreT/+*^*Gfi1*^*fl/fl*^ (red histogram and violin plots) NK cell chromatin accessibility and gene expression at *Cd226* gene locus as in (**a**). Grey box highlights a differentially accessible chromatin region. **j** Frequency of DNAM1^+^ NK cell subsets isolated from the spleen of naïve mice. Data shown as mean ± s.e.m. for one of three independent experiments (*n* = 6 mice). Statistical significance was calculated using a two-tailed Student’s *t* test (**c**, **e**, **g**, **h** and **j**).

GFI1-deficient Imm NK cells exhibited reduced expression of cyclin dependent kinases such as *Cdk1* and *Cdk2*, DNA replication machinery including *Mcm2, Mcm3 and Mcm4*, E2F family transcription factors and other cell cycle regulator genes (Supplementary Fig. 4a). Cell cycle pathway analysis showed reduced pathway enrichment in GFI1-deficient NK cells (Fig. 3a) that was associated with reduced *Mki67* chromatin accessibility (Fig. 3b). All GFI1-deficient NK cell subsets showed a reduction in Ki-67 expression suggesting reduced proliferation across all NK cell subsets in GFI1-deficient mice (Fig. 3c). Previously, we have shown that GFI1 is necessary to promote CD8^+^ T cell proliferation *via* E2F family of cell cycle regulators^18^. To ascertain whether GFI1 regulates NK cell proliferation *via* the E2F pathway, we quantified the E2F target gene enrichment score for cluster 11 (proliferating cells). This showed reduced enrichment in NK cells lacking GFI1 (Fig. 3d) and was accompanied by reduced *E2f1* and *E2f2* gene expression and chromatin accessibility (Supplementary Fig. 4b). These data argue that GFI1 promotes NK cell proliferation via epigenetic regulation of E2F proteins.

Given the change in genes affecting proliferation of GFI1-deficient NK cells, we investigated the ability of GFI1-deficient NK cells to respond to the cytokine IL-15 which supports NK cell survival and homeostatic proliferation^25^. Imm, M1 and M2 NK cells were purified from the spleen of naïve *Ncr*^*iCreT/+*^ and *Ncr*^*iCreT/+*^*Gfi1*^*fl/fl*^ mice, labelled with the cell tracker dye CTV and cultured in high (50 ng/mL) and low (25 ng/mL) concentrations of recombinant IL-15. This showed that very few GFI1-deficient NK cells entered cell division at higher concentrations of IL-15 and exhibited limited proliferative capacity when compared with *Ncr*^*iCreT/+*^ NK cells (Fig. 3e). Thus, GFI1 is required for IL-15-driven NK cell proliferation. Next, we examined genes involved in IL-15 signal transduction to test if reduced GFI1-deficient NK cell proliferation is due to defective IL-15 stimulation. IL-15 receptor genes such as *Il2rb*, *Il2rg* and *Il15ra* did not change in the absence of GFI1 (Fig. 3f), although *Stat5a* and *Stat5b* were reduced in GFI1-deficient NK cells (Supplementary Fig. 4c and Supplementary Data 1). Following IL-15 stimulation of NK cells, STAT5 phosphorylation was modestly reduced in NK cells lacking GFI1 compared with *Ncr*^*iCreT/+*^ NK cells (Fig. 3G). However, GFI1-tdTomato expression did not change following IL-15 treatment (Supplementary Fig. 4d), suggesting that GFI1 is not directly regulated by IL-15 signalling. Thus, IL-15 signalling remained largely intact, indicating that impaired proliferation reflects defects in cell-cycle control rather than cytokine responsiveness.

Next, we investigated whether GFI1 regulates NK cell activatory and inhibitory receptor expression. Among the Ly49 family of NK cell receptors, *Klra8*, which encodes Ly49H, was elevated in GFI1-deficient NK cells (Supplementary Fig. 4e). However, the frequency of Ly49H^+^ NK cells remained unchanged in *Ncr*^*iCreT/+*^*Gfi1*^*fl/fl*^ mice compared to *Ncr*^*iCreT/+*^ mice (Fig. 3h). DNAX accessory molecule-1 (DNAM-1) and T cell immunoreceptor with immunoglobulin and ITIM domains (TIGIT) are paired activating and inhibitory receptors that regulate NK cell effector function. In GFI1-deficient NK cells, expression of *Cd226* (DNAM-1) and *Tigit* was reduced (Supplementary Fig. 4e and Supplementary Data 1), accompanied by decreased chromatin accessibility at the *Cd226* and *Tigit* loci across both immature and differentiated NK cell states (Fig. 3i, Supplementary Fig. 4f, and Supplementary Data 3). Moreover, GFI1-deficient NK cells showed reduced expression of both DNAM-1 and TIGIT proteins at steady-state compared with wildtype NK cells (Fig. 3j and Supplementary Fig. 4g). Altogether, these data demonstrate that loss of GFI1 impacted NK cell proliferation and key NK cell surface receptors.

### GFI1 regulates NK cell cytotoxicity and promotes tumour control

**Fig. 4:**
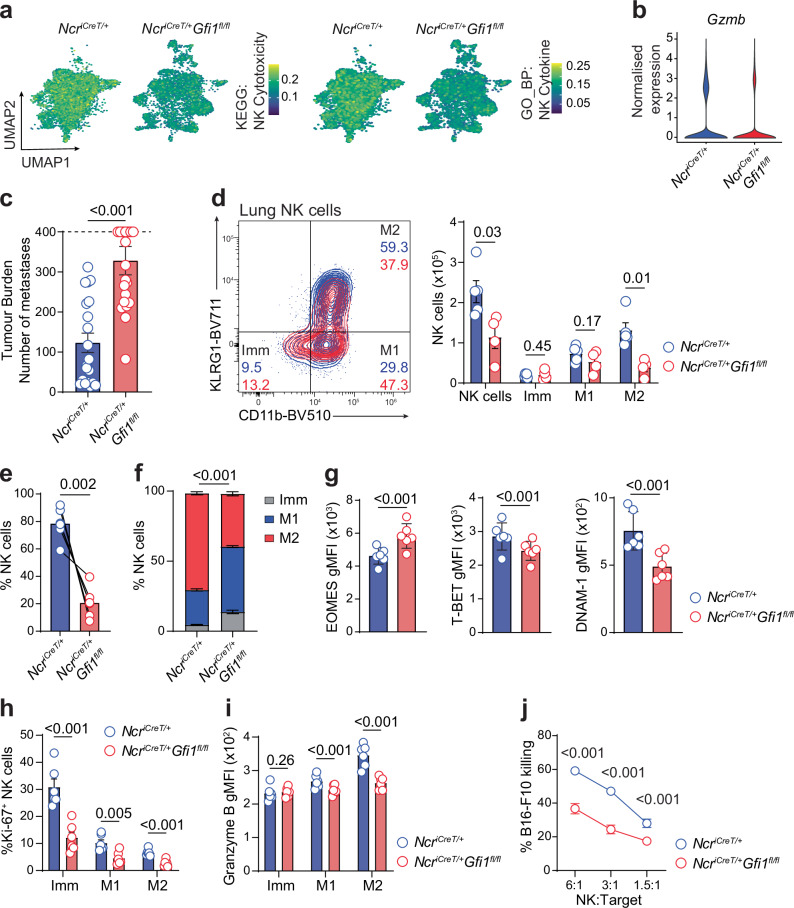
GFI1-deficient NK cells show impaired tumour metastasis control. **a** scMultiomic-seq data projected on UMAP showing NK cell cytotoxicity and NK cell cytokine pathway signature in NK cells isolated from the spleen of *Ncr*^*iCreT/+*^and *Ncr*^*iCreT/+*^*Gfi1*^*fl/fl*^ mice. **b** scMultiomic-seq based *Gzmb* expression in NK cells as in (**a**). **c** Number of tumour metastases in the lungs of *Ncr*^*iCreT/+*^and *Ncr*^*iCreT/+*^*Gfi1*^*fl/fl*^ mice 14 days after intravenous inoculation of B16-F10 tumour cells. The dashed line represents the upper detection limit. Data pooled from three independent experiments shown as mean ± s.e.m. (*n* = 17 or 19 mice/genotype). **d** Representative plot (left) and quantification (right) of NK cell subset frequency in lungs of *Ncr*^*iCreT/+*^ and *Ncr*^*iCreT/+*^*Gfi1*^*fl/fl*^ mice at day 14 after tumour inoculation. Data from one of the two independent experiments shown as mean ± s.e.m. (*n* = 4 *Ncr*^*iCreT/+*^; 5 *Ncr*^*iCreT/+*^*Gfi1*^*fl/fl*^ mice). **e** Frequency of *Ncr*^*iCreT/+*^ (CD45.1^+^CD45.2^+^) and *Ncr*^*iCreT/+*^*Gfi1*^*fl/fl*^ (CD45.2^+^) NK cells among total donor NK cells in the lungs of chimeric mice (CD45.1^+^) that received B16-F10 cell intravenously 6 weeks after bone marrow reconstitution, and lungs were isolated 14 days after tumour inoculation. **f** Frequency of Imm, M1 and M2 NK cell subsets isolated from the lungs of bone marrow chimeric mice as in (**e**). **g** EOMES, T-BET and DNAM-1 expression levels in lung NK cells as in (**e**). **h** Frequency of Ki-67^+^ cells among NK cell subsets isolated from lungs as in (**e**). **i** Granzyme B expression in NK cell subsets isolated from lungs as in (**e**). NK cells were stimulated with PMA + ionomycin for 4 hours before analysis. **e**–**i** Data pooled from two independent experiments shown as mean ± s.e.m. (*n* = 6 mice). **j** In vitro killing of B16-F10 target cells by splenic NK cells at the indicated NK:effector ratios. Data from one of the two independent experiments shown as mean ± s.e.m. (*n* = 5-6 mice/genotype). Statistical significance was calculated using a two-tailed Mann-Whitney U test (**c**), a two-tailed Student’s *t* test (**d** and **j**) or a two-tailed paired *t* test (**e**–**i**).

In cancer, NK cells are responsible for mediating protection through inflammatory cytokines and cytotoxic molecules that kill tumour cells^26^. Pathway enrichment analysis using scRNA-seq data showed NK cell cytotoxicity and NK cell cytokine production pathways were both downregulated in NK cells lacking GFI1 compared to wildtype NK cells (Fig. 4a). Analysis of NK cell function showed that ablation of GFI1 in NK cells resulted in diminished IFN-γ production in response to a stimulus, especially in differentiated M1 and M2 NK cells (Supplementary Fig. 5a). Moreover, *Gzmb* gene expression (Fig. 4b) and chromatin accessibility (Supplementary Fig. 5b) was reduced in *Ncr*^*iCreT/+*^*Gfi1*^*fl/fl*^ NK cells suggesting that GFI1 controlled NK cell cytotoxicity. Thus, to investigate whether the absence of GFI1 affected the capacity of NK cells to control tumour, *Ncr*^*iCreT/+*^ and *Ncr*^*iCreT/+*^*Gfi1*^*fl/fl*^ mice were inoculated with 2×10^5^ B16-F10 murine melanoma cells *via* tail vein injection and the development of lung metastasis was tracked. Although GFI1-sufficient NK cells largely controlled the B16-F10 melanoma as evidenced by low tumour burden, GFI1-deficient NK cells were unable to prevent the development of tumours in lungs, resulting in higher tumour burden than controls (Fig. 4c and Supplementary Fig. 5c). This limited control was paralleled by the detection of fewer NK cells in the lung and spleen, particularly M2 NK cells, in tumour-bearing GFI1-deficient mice (Fig. 4d and Supplementary Fig. 5d). To understand the factors influencing this lack of tumour control, mixed bone marrow chimeras were generated that were challenged with B16-F10 cells, six weeks after BM reconstitution. In this setting, GFI1-deficient NK cells were significantly outcompeted by their wildtype counterparts in lungs and spleen of tumour-bearing chimeric mice (Fig. 4e and Supplementary Fig. 5d). Among GFI1-deficient lung NK cells of chimeric mice, fewer than 40% were M2 NK cells compared with 75% M2 NK cells among wildtype NK cells (Fig. 4f). Lung NK cells isolated from tumour-bearing chimeric mice showed reduced frequency of GFI1-deficient NK cells (Supplementary Fig. 5e), with elevated EOMES and reduced T-BET expression in NK cell lacking GFI1 (Fig. 4g). Moreover, chimeric mice showed reduced DNAM-1 and TIGIT expression (Fig. 4g and Supplementary Fig. 5f) and impaired NK cell proliferation in GFI1^–/–^ NK cells (Fig. 4h). Loss of GFI1 also impaired IFN-γ and granzyme B production from NK cells isolated from tumour-bearing lungs (Fig. 4i and Supplementary Fig. 5g). As GFI1 deficiency impaired both NK cell proliferation and NK cell cytotoxic molecule production, we performed an in vitro B16-F10 killing assay with equal number of control and GFI1-deficient NK cells to ascertain whether impaired tumour control in *Ncr*^*iCreT/+*^*Gfi1*^*fl/fl*^ mice is due to reduced NK cell number, impaired cytotoxicity or both. In vitro killing assay demonstrated that the lack of GFI1 severely reduced NK cell killing capacity (Fig. 4j), in line with reduced cytotoxic effector molecule production. Together, these data demonstrate that intrinsic GFI1 expression in NK cells regulates NK cell cytotoxicity and tumour control capacity.

### GFI1 expression is required for antiviral NK cell responses

**Fig. 5:**
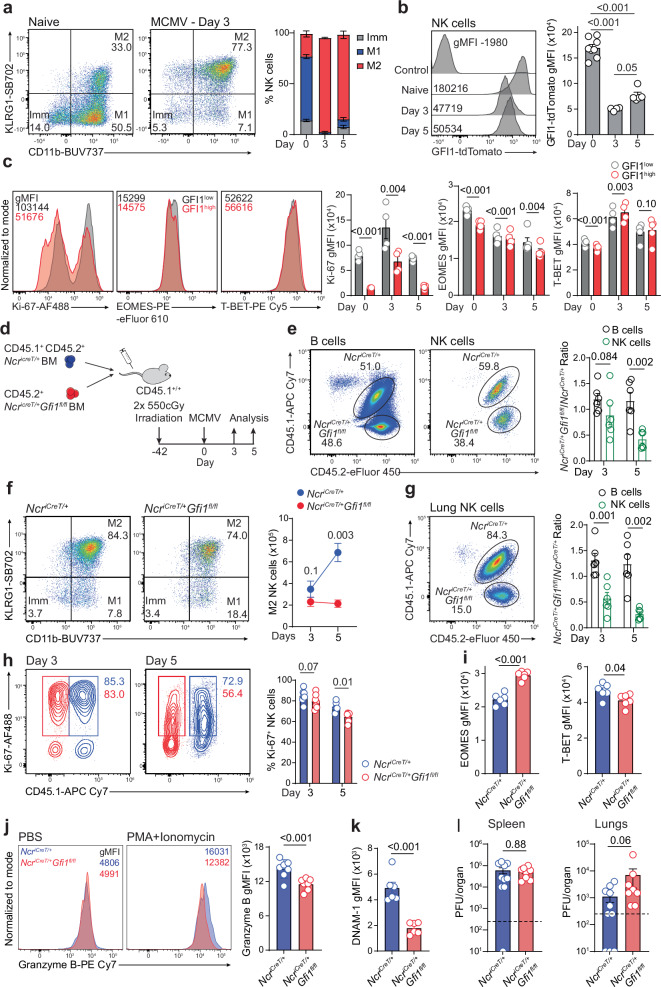
Loss of GFI1 impairs NK cell proliferation and activation following virus infection. **a** Flow cytometric dot plots (left) and bar plots (right) showing the frequency of NK cell subsets isolated from the spleen of *Gfi1*^*tdTomato*/+^ mice infected with 2 × 10^5^ PFU of MCMV intraperitoneally. **b** Histograms (left) and gMFI quantification (right) of GFI1-tdTomato expression in NK cells isolated from the spleen as in (**a**). **c** Histograms showing Ki-67, EOMES and T-BET expression in GFI1high (red) and GFI1low (grey) NK cells isolated from the spleen of *Gfi1*^*tdTomato/+*^ mice at 3 days after infection (left panels). Bar plots show Ki-67, EOMES and T-BET gMFI as mean ± s.e.m. (right panels). **a**–**c** Data pooled from two independent experiments and show mean ± s.e.m. (*n* = 4, 5 or 8 mice/ timepoint). **d** Schematic showing bone marrow chimeric mice generation, MCMV infection (2×10^5^ PFU) and analysis timeline. **e** Flow cytometric dot plots showing frequency of splenic B and NK cells derived from donor *Ncr*^*iCreT/+*^ (CD45.1^+^CD45.2^+^) and *Ncr*^*iCreT/+*^*Gfi1*^*fl/fl*^ (CD45.2^+^) bone marrow at 3 days after MCMV infection of chimeric mice (CD45.1^+^), 8 weeks after bone marrow reconstitution (left). Ratio of *Ncr*^*iCreT/+*^ to *Ncr*^*iCreT/+*^*Gfi1*^*fl/fl*^ cells in B and NK cell populations from the spleen after infection (right panel). **f** Flow cytometric dot plots showing frequency of Imm, M1 and M2 NK cells from the spleen as in D (left). Total number of M2 (CD11b^+^KLRG1^+^) NK cells isolated from the spleen after infection (right). **g** Dot plot showing frequency of NK cells in lungs of chimeric mice at 3 days after infection (left). Ratio of *Ncr*^*iCreT/+*^ to *Ncr*^*iCreT/+*^*Gfi1*^*fl/fl*^ cells in B and NK cell populations from the lungs as in (**e**) (right). **h** Flow cytometric plots (left) and quantification (right) showing frequency of Ki-67^+^ cells among total *Ncr*^*iCreT/+*^ and *Ncr*^*iCreT/+*^*Gfi1*^*fl/fl*^ NK cells after infection as in (**e**). **i** gMFI of EOMES and T-BET in splenic NK cells isolated at 3 days after infection, as in (**e**). **j** Histogram (left) and gMFI quantification (right) of Granzyme B expression in splenic *Ncr*^*iCreT/+*^ (blue lines) and *Ncr*^*iCreT/+*^*Gfi1*^*fl/fl*^ (red lines) NK cells at day 3 after infection, as in (**e**) and stimulated with PMA + ionomycin in vitro for 4 hours. **k** DNAM-1 expression in NK cells isolated from the spleen of chimeric mice at day 3 after infection, as in (**e**). **e**–**k** Data pooled from two independent experiments are shown as mean ± s.e.m. (*n* = 6 mice/timepoint). **l** Virus replication in spleen and lungs of *Ncr*^*iCreT/+*^ and *Ncr*^*iCreT/+*^*Gfi1*^*fl/fl*^ mice euthanized at day 3 after infection with 1 × 10^6^ PFU of MCMV is measured using plaque- forming assay and shown as mean ± s.e.m. Broken line represents assay detection limit. Data pooled from two independent experiments (*n* = 8 or 10 mice/genotype). Statistical significance was calculated using one-way ANOVA and Tukey’s post-test (**b**), a two-tailed paired Student’s *t* test (**c**,**e**–**k**) or a two-tailed Mann-Whitney U test (**l**).

To investigate how GFI1 is regulated in NK cells following virus infection, *Gfi1*^*tdTomato/+*^ reporter mice were challenged with murine cytomegalovirus (MCMV) that is known to induce strong NK cell responses^27,28^. This induced strong NK cell maturation characterised by upregulation of CD11b, CD62L, KLRG1 and DNAM-1 while CD27 and CD127 were downregulated (Fig. 5a and Supplementary Fig. 6a). This MCMV-induced maturation^29^ was parallelled by significant GFI1-tdTomato downregulation in all NK cell subsets that was maintained at day 3 and 5 (Fig. 5b and Supplementary Fig. 6b). Following virus infection, GFI1^low^ NK cells showed higher Ki-67 and EOMES expression than GFI1^high^ NK cells, while T-BET expression was slightly higher in GFI1^high^ NK cells at day 3 after infection (Fig. 5c). Next, we analysed the anti-viral response of *Ncr*^*iCreT/+*^ and *Ncr*^*iCreT/+*^*Gfi1*^*fl/fl*^ NK cells in mixed bone marrow chimeras (Fig. 5d). In peripheral blood, the frequency of GFI1-deficient NK cells remained lower after infection (Supplementary Fig. 6c). While the prevalence of *Ncr*^*iCreT/+*^*Gfi1*^*fl/fl*^ NK cells was similar to *Ncr*^*iCreT/+*^ NK cells in the spleen at day 3 after infection, this decreased at day 5 after infection (Fig. 5e). The frequency and total number of GFI1-deficient M2 cells was lower than GFI1 competent cells at day 5 after infection in the spleen (Fig. 5f) and lungs (Fig. 5g). Both NK cell populations had a similar frequency of Ki-67^+^ cells at day 3 after infection, however GFI1-deficient NK cells showed lower Ki-67 expression by day 5 after infection (Fig. 5h). Collectively, these data show that intrinsic GFI1 expression was required to maintain NK cell terminal maturation and proliferation following virus infection.

Previously, MCMV-induced maturation of NK cells has been shown to be associated with downregulation of EOMES in immunocompetent animals^29^. GFI1-deficient NK cells showed an increase in EOMES expression after virus infection in spleen and lungs, while T-BET expression was reduced (Fig. 5i and Supplementary Fig. 6d, e). Loss of GFI1 also impaired granzyme B production and DNAM-1 expression (Fig. 5j, k). These findings suggest that GFI1 expression is required for maintaining NK cell responses following virus infection. MCMV virus replication was measured in *Ncr*^*iCreT/+*^ and *Ncr*^*iCreT/+*^*Gfi1*^*fl/fl*^ mice to determine if virus control was impaired. However, virus control was similar in the spleen and lungs (Fig. 5l). Thus, NK cell cytotoxicity alone does not appear to be essential for virus control that can be redundantly controlled by other immune cell populations such as conventional CD8^+^ T cells and γδ T cells^30^.

### Loss of EOMES fails to rescue GFI1-deficient NK cell maturation

EOMES is required for early NK cell development but is downregulated during terminal maturation^31^. Consistent with this stage-specific role, GFI1-deficient NK cells exhibited elevated EOMES expression at steady-state (Fig. 1i) and following inflammatory challenge (Fig. 5h). To test whether increased EOMES contributed to the maturation defect observed in the absence of GFI1, mice lacking both GFI1 and EOMES in NK cells (*Ncr*^*iCreT/+*^*Eomes*^*fl/fl*^*Gfi1*^*fl/fl*^ mice) were analysed. Deletion of either GFI1 or EOMES alone resulted in a reduction of M2 NK cell frequency in the spleen, while combined deletion resulted in a severe reduction of total NK cells in the spleen and BM, and a marked failure in the generation of M1 and M2 NK cell subsets (Supplementary Fig. 7a–d). Thus, removal of EOMES did not rescue, but instead exacerbated the NK cell developmental defects caused by GFI1-deficiency.

To assess whether partial reduction of EOMES might alleviate GFI1-dependent defects, we analysed GFI1-deficient NK cells heterozygous for EOMES (*Ncr*^*iCreT/+*^*Eomes*^*fl/+*^*Gfi1*^*fl/fl*^). EOMES haploinsufficiency failed to restore NK cell numbers or maturation (Supplementary Fig. 7e), likely due to the critical role of EOMES in early bone marrow NK cell precursors^32^. Together, these data indicate that although GFI1 normally restrains EOMES expression during later stages of NK cell differentiation, EOMES remains indispensable for early NK cell development and cannot substitute for GFI1-dependent regulatory programmes.

### GFI1-FOXO1 axis controls NK cell proliferation and differentiation

**Fig. 6:**
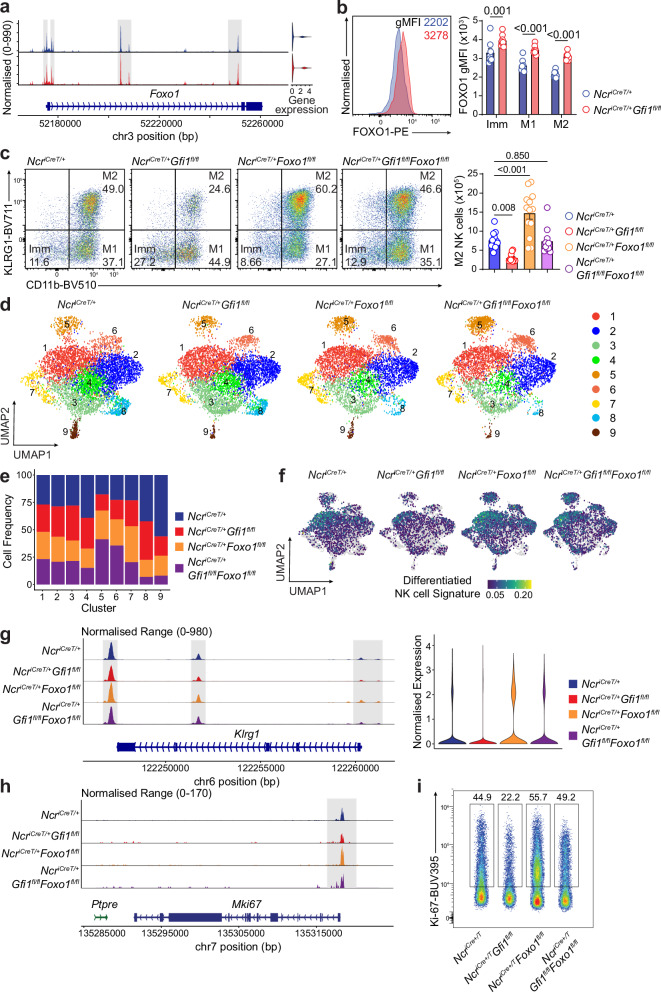
GFI1 mediated FOXO1 repression is important for NK cell differentiation and proliferation. **a** scMultiomic-seq data showing *Foxo1* chromatin accessibility and gene expression in total *Ncr*^*iCreT/+*^ (blue histogram and violin plots) and *Ncr*^*iCreT/+*^*Gfi1*^*fl/fl*^ (red histogram and violin plots) NK cells isolated from the spleen of naïve mice. Grey boxes highlight differentially accessible chromatin regions. **b** Histograms (left) and gMFI quantification (right) of FOXO1 expression in splenic NK cells isolated from naïve mice. Data pooled from two independent experiments are shown as mean ± s.e.m. (*n* = 8 mice/genotype). **c** Representative plots showing frequency of NK cell subsets isolated from spleen of naïve *Ncr*^*iCreT/+*^*, Ncr*^*iCreT/+*^*Gfi1*^*fl/fl*^*, Ncr*^*iCreT/+*^*Foxo1*^*fl/fl*^ *and Ncr*^*iCreT/+*^*Gfi1*^*fl/fl*^*Foxo1*^*fl/fl*^ mice (left). Frequency of M2 NK cells across the different genotypes is shown as mean ± s.e.m. (right). Data pooled from two independent experiments (*n* = 10 or 13 mice/genotype). **d** UMAP shows scRNA-seq based unsupervised clustering of splenic *Ncr*^*iCreT/+*^ (*n* = 6225 cells), *Ncr*^*iCreT/+*^*Gfi1*^*fl/fl*^ (*n* = 5654 cells), *Ncr*^*iCreT/+*^*Foxo1*^*fl/fl*^ (*n* = 4909) and *Ncr*^*iCreT/+*^*Gfi1*^*fl/fl*^*Foxo1*^*fl/fl*^ (*n* = 4839) NK cells isolated for scMultiomic-seq from naïve mice. **e** Frequency of NK cells in each cluster identified in panel (**d**). **f** UMAP showing differentiated NK cell gene expression signature enrichment as in (**d**). **g** *Klrg1* chromatin accessibility (left) and gene expression (right) in splenic NK cells as in (**d**). Grey boxes highlight differentially accessible chromatin regions. **h** *Mki67* chromatin accessibility in splenic NK cell loci as in (**d**). Grey boxes highlight differentially accessible chromatin regions. **i** Frequency of Ki-67^+^ NK cells isolated from the spleen of naïve mice. Concatenated data are representative of two independent experiments (*n* = 4 or 5 mice/genotype). Statistical significance was calculated using a two-tailed Student’s *t* test (**b**) or a one-way ANOVA with Dunnett’s post-test (**c**).

Previous studies have shown that FOXO1 is a suppressor of NK cell maturation and function^7,33^, but how FOXO1 is regulated in NK cells is not known. The scMultiomic-seq analysis showed that GFI1-deficient NK cells upregulated *Foxo1* gene expression along with increased chromatin accessibility (Fig. 6a and Supplementary Fig. 8a). Analysis of FOXO1 protein showed that it was significantly elevated in all GFI1^–/–^ NK cell subsets (Fig. 6b). This suggests that GFI1-mediated FOXO1 suppression may regulate NK cell maturation and function. To test this, both *Gfi1* and *Foxo1* were genetically ablated in NK cells by generating *Ncr*^*iCreT/+*^*Gfi1*^*fl/fl*^*Foxo1*^*fl/fl*^ double knockout (DKO) mice. While the loss of FOXO1 alone resulted in an increase in the prevalence of M2 NK cells, the combined loss of FOXO1 and GFI1 reduced the frequency of M2 NK cells to that of *Ncr*^*iCreT/+*^ NK cells (Fig. 6c). The NK cell and M2 cell number in spleen, lungs and liver of *Ncr*^*iCreT/+*^*Gfi1*^*fl/fl*^*Foxo1*^*fl/fl*^ mice was restored to the level of *Ncr*^*iCreT/+*^ (wildtype) mice, while the number of FOXO1-deficient NK cells was increased in these tissues (Supplementary Fig. 8b, c).

To understand the epigenetic and transcriptional regulatory networks regulated by the GFI1-FOXO1 axis or GFI1 alone, splenic NK cells were purified from naïve *Ncr*^*iCreT/+*^*Foxo1*^*fl/fl*^ and *Ncr*^*iCreT/+*^*Foxo1*^*fl/fl*^*Gfi1*^*fl/fl*^ mice, and scMultiomic-seq was performed. The scRNA-seq data from FOXO1-deficient and DKO NK cells were then integrated with our GFI1-deficient and wild-type (*Ncr*^*iCreT/+*^) NK cell datasets using reciprocal principal component analysis to perform unsupervised clustering. NK cells segregated into 9 distinct clusters spanning across the 4 genotypes (Fig. 6d). Clusters 1 was composed of differentiated NK cells exhibiting highest levels of *Gzma*, *Itgam* and *Zeb2* expression, together with low *Cd27* and *Il7r* expression (Supplementary Fig. 8d). The four NK cell genotypes were equally represented in cluster 1, with *Ncr*^*iCreT/+*^, *Ncr*^*iCreT/+*^*Gfi1*^*fl/fl*^, *Ncr*^*iCreT/+*^*Foxo1*^*fl/fl*^ and *Ncr*^*iCreT/+*^*Foxo1*^*fl/fl*^*Gfi1*^*fl/fl*^ NK cells comprising 27.8%, 24.8%, 24.1% and 23.3% of total cells in cluster 1, respectively (Fig. 6e). Cluster 8 represented the immature NK cells defined by high *Il7r* and *Tcf7* expression and lower levels of *Klrg1*, *Itgam* and *Gzma* expression. FOXO1-deficient and DKO NK cells were under-represented in this clusters (Supplementary Data 4). In contrast, NK cells lacking FOXO1, alone or together with GFI1, were enriched in clusters 5 and 6. These clusters represented differentiated NK cells, similar to cluster 1, with high levels of *Gzma, Klrg1* and *Zeb2* expression (Supplementary Fig. 8d). Notably, cluster 5 was distinguished from cluster 1 by higher *Klrg1* expression (Supplementary Data 5). The NK cell differentiation signature was highly enriched in FOXO1-deficient NK cell transcriptome, while the lowest score was observed in cells lacking GFI1 (Fig. 6f). Notably, NK cells lacking both GFI1 and FOXO1 exhibited a differentiation signature comparable to wild-type NK cells. *Klrg1* and *Itgam* expression was rescued in DKO NK cells (Fig. 6g and Supplementary Fig. 8e), likely due to increased chromatin accessibility of the *Klrg1* locus. Thus, GFI1 and FOXO1 co-regulate NK cell maturation and expression of key NK cell receptors. Although the frequencies of *Ncr*^*iCreT/+*^*Foxo1*^*fl/fl*^ and *Ncr*^*iCreT/+*^*Gfi1*^*fl/fl*^*Foxo1*^*fl/fl*^ NK cells in clusters 9, annotated as proliferating cells due to high expression of cell cycle related genes such as *Mki67*, *Top2a* and *E2f2*, were reduced as compared to *Ncr*^*iCreT/+*^ NK cells, FOXO1-deficient and DKO NK cells showed higher *Mki67* chromatin accessibility than NK cells lacking GFI1 (Fig. 6h). FOXO1-deficeint cells exhibited higher frequency of Ki-67^+^ NK cells in spleen, whereas combined loss of both GFI1 and FOXO1 restored Ki-67^+^ NK cell frequency to levels comparable to *Ncr*^*iCreT/+*^ NK cells (Fig. 6i). Together, these data position GFI1 and FOXO1 as opposing regulators that converge on shared chromatin programmes to balance NK cell maturation, proliferation and effector function.

### FOXO1-driven NK cell molecular programmes

FOXO1 has been shown to supress NK cell differentiation^7^, yet the downstream molecular pathways regulated by FOXO1 remain undefined. We focused on the analysis of FOXO1-deficient NK cells to define the molecular pathways regulated by FOXO1 in NK cells. Neighbourhood analysis showed that NK cells lacking FOXO1, either alone or in combination with GFI1 loss, were enriched in cluster 5, 6 and 7, with a corresponding depletion in cluster 8 (immature NK cells) and 9 of integrated datasets (Supplementary Fig. 9a). Cluster 5 and 6 were composed of differentiated NK cells (Supplementary Fig. 9b) and FOXO1-deficient NK cell enrichment in these clusters was aligned with the known role of FOXO1 to suppress NK cell differentiation^33^. FOXO1-deficient NK cells showed elevated levels of transcription factors that promote NK cell differentiation, such as *Zeb2* and *Tbx21*, alongside reduced levels of *Tcf7*, *Id2* and *Eomes* (Supplementary Fig. S9B). Loss of FOXO1 resulted in elevated *Itgam*, *Klrg1* and *Sell* expression, while *Itgax* (CD11c) was reduced compared to wildtype NK cells. The expression of cytotoxic effector molecules such as *Gzma*, *Gzmb* and *Prf1* increased in the absence of FOXO1, which aligns with the critical role of FOXO1 in regulating NK cell killing capacity. Cell-cycle and proliferation regulating genes such as *E2f1* and *E2f2* were elevated in the absence of FOXO1 (Supplementary Fig. 9b). These transcriptional changes were associated with corresponding epigenetic changes such as open chromatin at *Tbx21*, *Zeb2* and *Klrg1* loci (Supplementary Fig. 9c). *Foxo1* expression itself increased in the absence of functional FOXO1 (Supplementary Fig. 3c), suggesting self-regulation of its transcription. Most FOXO1-dependent DEGs exhibited reciprocal regulation relative to GFI1, but some genes, including *Ccr2, Jun* and *Fosb*, displayed FOXO1-specific regulation that was not mirrored by GFI1 (Supplementary Fig. 9c and Supplementary Data 6). Together, these data indicate that while GFI1 and FOXO1 co-regulate many targets, GFI1 also independently controls a broader NK cell gene programme. Consistent with this model, a substantially larger number of genes were regulated by GFI1 alone compared with FOXO1 (4934 DEGs in *Ncr*^*iCreT/+*^*Gfi1*^*fl/fl*^ versus 638 DEGs in *Ncr*^*iCreT/+*^*Foxo1*^*fl/fl*^ relative to *Ncr*^*iCreT/+*^ NK cells), including genes such as *Nt5e* and *Sgk1* that were elevated in GFI1-deficient and DKO NK cells but remain unchanged in FOXO1-deficient cells (Supplementary Data 1 and 6). Notably, the molecular expression patterns of FOXO1-deficient NK cells were reciprocal to those observed in GFI1-deficent NK cells, suggesting that NK cell transcriptional programmes that control NK cell proliferation, differentiation and effector function are controlled by opposing actions of GFI1 and FOXO1.

### Combined loss of GFI1 and FOXO1 partially restores NK cell tumour protection

**Fig. 7:**
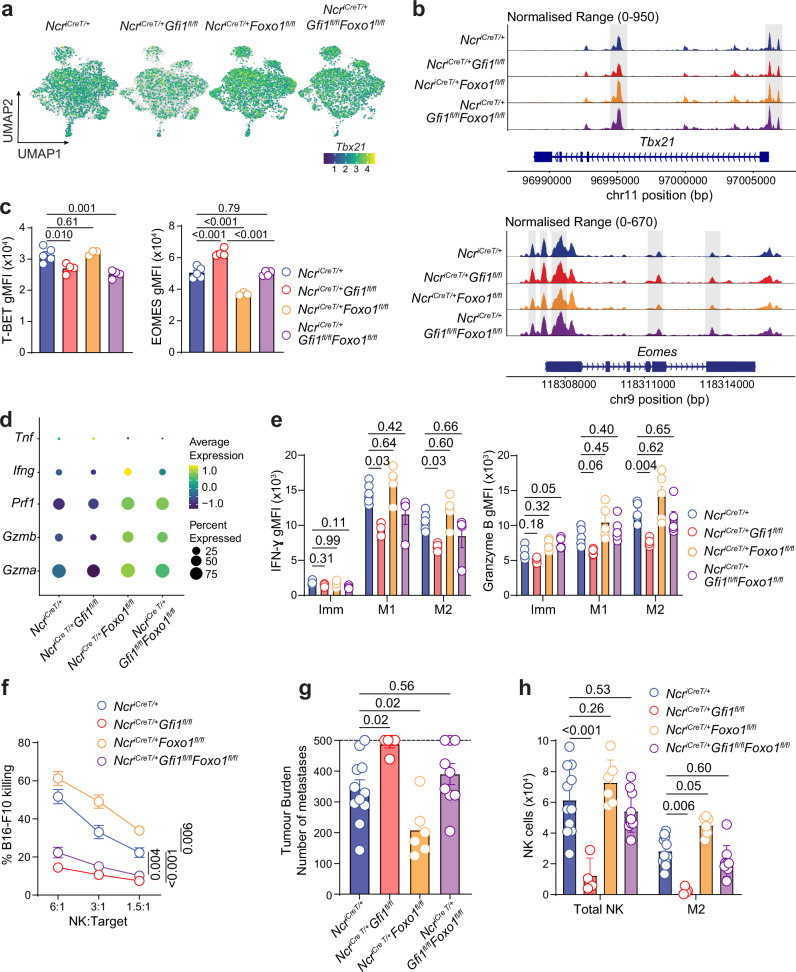
Genetic abrogation of FOXO1 restores GFI1-deficient NK cell tumour control. **a** UMAP showing *Tbx21* gene expression in splenic NK cells isolated for scMultiomic-seq from the spleen of naïve mice. **b** *Tbx21* (top) and *Eomes* (bottom) chromatin accessibility in total splenic NK cells as in (**a**). Grey boxes highlight differentially accessible chromatin regions. **c** Quantification of T-BET and EOMES expression in splenic NK cells isolated from naïve mice. Data from one of three experiments are shown as mean ± s.e.m. (*n* = 3, 4 or 5 mice/genotype/experiment). **d** Dot plots showing expression of cytotoxicity related gene in splenic NK cells as in (**a**). **e** IFN- γ and Granzyme B expression in splenic NK cell subsets isolated from naïve mice after 4 h in vitro stimulation with PMA + ionomycin. Data from one of two independent experiments is shown as mean ± s.e.m. (*n* = 4, 5 or 6 mice/genotype/experiment). **f** In vitro killing of B16-F10 target cells by splenic NK cells at the indicated NK:effector ratios. Data from one of the two independent experiments shown as mean ± s.e.m. (*n* = 4 mice/ genotype/experiment). **g** Number of tumour metastases in mice lungs 14 days after intravenous inoculation of B16-F10 tumour cells. The dashed line represents the upper detection limit. Data pooled from two independent experiments shown as mean ± s.e.m. (*n* = 6, 9 or 11 mice/genotype). **h** Number of total and M2 NK cells isolated from lungs at 14 days after tumour inoculation. Data pooled from two independent experiments shown as mean ± s.e.m. (*n* = 4, 6, 9 or 11 mice/genotype). Statistical significance was calculated using a one-way ANOVA and Tukey’s post-test (**c** and **g**) or a two-way ANOVA test with Dunnett’s post-test (**e**, **f** and **h**).

Given that the GFI1-FOXO1 axis regulated NK cell maturation and proliferation, and that GFI1- and FOXO1-driven molecular programmes were reciprocal, we analysed scMultiomic-seq data to identify gene co-regulated by GFI1 and FOXO1. Comparison of the transcriptional and epigenetic profiles of *Ncr*^*iCreT/+*^*Gfi1*^*fl/fl*^*Foxo1*^*fl/fl*^ and *Ncr*^*iCreT/+*^*Gfi1*^*fl/fl*^ NK cells using pseudobulk analysis showed significant upregulation of NK cell differentiation-associated genes, such as *Tbx21* and *Zeb2*, in the double knockout, together with increased chromatin accessibility at these loci (Fig. 7a and Supplementary Fig. 10a). *Eomes* expression was significantly reduced in DKO NK cells compared with GFI1-deficient NK cells (Supplementary Fig. 10b and Supplementary Data 6) while chromatin accessibility at *Eomes* and *Tbx21* loci were restored in NK cells lacking both GFI1 and FOXO1 (Fig. 7b). However, only EOMES protein expression was restored to wildtype levels in DKO NK cells, whereas T-BET expression remained lower in *Ncr*^*iCreT/+*^*Gfi1*^*fl/fl*^*Foxo1*^*fl/fl*^ NK cells (Fig. 7c). This indicated that GFI1 regulated T-BET expression independently of FOXO1, while EOMES expression was regulated *via* FOXO1.

Loss of FOXO1 in GFI1-deficient NK cells substantially restored expression of NK cell genes involved in differentiation and function (Supplementary Fig. 10b). We next investigated whether the combined loss of GFI1 and FOXO1 was sufficient to rescue NK cell cytotoxicity and cytokine production. Indeed, loss of FOXO1 in GFI1-deficient cells recovered *Gzma*, *Gzmb*, and *Ifng* transcript expression and restored IFN-γ and granzyme B protein production to wild-type levels (Fig. 7d, e). Given this, we next quantified the killing capacity of DKO NK cells. While NK cell lacking FOXO1 killed B16-F10 target cells significantly more efficiently than wildtype NK cells, combined loss of FOXO1 and GFI1 led to a modest, but not statistically significant, increase (54.3%) in NK cell killing capacity compared with GFI1-deficient NK cells (Fig. 7f). Analysis of genes involved in NK cell degranulation, including lysosomal transport, SNARE components and cytoskeleton machinery, showed that combined loss of GFI1 and FOXO1 did not restore cytoskeleton and immune synapse related genes such as *Rac2*, *Coro1a* and *Cfl1* (Supplementary Fig. 10c). These findings show that while loss of FOXO1 largely rescued effector molecule production in GFI1-deficient NK cells, full restoration of cytotoxic function requires additional GFI1-dependent pathways.

Next, we investigated whether the anti-tumour functions of GFI1-deficient NK cells were restored by FOXO1 ablation. *Ncr*^*iCreT/+*^*Foxo1*^*fl/fl*^ mice had a lower number of tumour metastasis than wildtype mice (Fig. 7g and Supplementary Fig. 11a). In contrast, mice lacking both GFI1 and FOXO1 developed a tumour burden comparable with wildtype mice (Fig. 7g). Paralleling these responses, the combined loss of GFI1 and FOXO1 in NK cells rescued NK cell number and the development of M1 and M2 subsets in lungs of tumour-bearing mice (Fig. 7h and Supplementary Fig. 11b). The improved tumour control and increase in NK cells in *Ncr*^*iCreT/+*^*Gfi1*^*fl/fl*^*Foxo1*^*fl/fl*^ mice was associated with improved proliferation (Ki-67 expression) and DNAM-1 expression in Imm NK cells that give rise to M1 and M2 cells compared with GFI1-deficient Imm NK cells (Supplementary Fig. 11c, d). Altogether, these data demonstrate that although the combined loss of GFI1 and FOXO1 did not fully restore NK cell killing capacity, the recovery of NK cell differentiation and proliferation was sufficient to provide effective protection against tumour metastasis.

## Discussion

In this study, we identify GFI1 as a central, cell-intrinsic regulator of NK cell differentiation and effector function. By integrating transcriptional and epigenetic programmes that govern maturation, proliferation and receptor acquisition, GFI1 enables the generation of terminally differentiated NK cells capable of effective tumour control. Unlike transcription factors that primarily promote or restrain discrete stages of NK cell differentiation, GFI1 coordinates multiple regulatory layers, including chromatin accessibility, transcription factor balance and proliferative capacity to establish the NK cell differentiation programme. GFI1 epigenetically repressed genes such as *Eomes* and *Foxo1* necessary to regulate NK cell activatory receptor expression and effector functions important for controlling tumour metastasis. In addition, we show that the GFI1-FOXO1 axis is a key regulatory pathway that fine-tunes NK cell maturation and functional capacity by regulating effector molecule production. Altogether, we demonstrate that GFI1 is required for progression from immature to mature NK cell states through a previously unrecognised GFI1-FOXO1 axis and that its loss disrupts the balance of key transcription factors that orchestrate NK cell fate and function essential for NK cell–mediated immunity.

GFI1 is highly expressed in hematopoietic stem cells, common lymphoid progenitors and T cells, and it regulates cell cycle and self-renewal capacity in these cells^15,18,20^. However, its role in mature NK cells and stage-specific requirements in peripheral NK cell development has not been previously elucidated. Our data support a model in which GFI1 shapes the NK cell chromatin landscape to coordinate differentiation with proliferative and effector programmes. NK cells lacking GFI1 were transcriptionally and epigenetically skewed toward an immature state with reduced M1 and M2 subsets across peripheral tissues. Specific deletion of GFI1 in NKp46-expressing NK cells resulted in a significant defect in multiple NK cell regulatory pathways, revealing a global rewiring of transcription factors that were de-repressed in the absence of GFI1. This uncovered two pathways, EOMES and FOXO1, that were co-regulated by GFI1, but each pathway was uniquely regulated. It was also revealed that loss of GFI1 alone disrupted the responsiveness of NK cells to IL-15-driven amplification, resulting in poor survival and proliferation of NK cells. Furthermore, it affected multiple inhibitory, activatory and homing molecules, potentially resulting in a redistribution of NK cells across tissues and a failure of the normal NK cells immunosurveillance programme. This revealed that rather than GFI1 acting as a lineage-specific switch, GFI integrates multiple regulatory inputs to enforce the coordinated execution of NK cell differentiation programmes necessary for mature NK cell development and homoeostasis.

It is well established that EOMES and T-BET are essential for NK cell development, and the balance between these two factors is critical for NK cell maturation and activation^34^. EOMES and T-BET have generally been considered to function independently. Yet despite clear evidence that their relative expression levels are critical, the molecular mechanisms that establish and maintain this balance are poorly defined. Furthermore, how other transcription factors influence their function has not been well understood. Here, we show that EOMES expression in NK cells is increased in the absence of GFI1. Complete loss of EOMES resulted in significantly impaired Imm NK cell development, likely through enhanced apoptosis^34,35^, while GFI1-deficient Imm NK cells were not significantly impacted. However, combined deletion of EOMES and GFI1 failed to rescue NK cell development and function, but instead further exacerbated the defects. EOMES itself is not a repressor and is essential for NK cell maturation and functional specification^32,36,37^. The level of EOMES expression is finely tuned relative to T-BET. However, how this precise level is regulated remains unclear. From our data, we would posit that GFI acts to calibrate EOMES expression to establish the EOMES–T-BET balance necessary for NK cell specification. A similar dichotomy has been observed for GFI1, which regulates TH2 and TH17 effector programmes and thereby shapes the balance of cytokines produced by CD4^+^ T cell subsets^16^.

In contrast to the loss of EOMES in GFI1-deficient NK cells, loss of FOXO1 resulted in a substantial, albeit partial, rescue of the GFI1 phenotype. FOXO1 has been identified as a suppressor of NK cell activation through the regulation of T-BET^7^. Our findings identify FOXO1 as a key downstream mediator of GFI1-dependent regulation and establish a GFI1–FOXO1 axis that integrates opposing transcriptional cues to regulate NK cell differentiation. FOXO1 has been previously proposed to regulate NK cell maturation *via* T-BET, a conclusion drawn from studies of *Ncr1*^*iCre*^*Foxo1*^*fl/fl*^*Tbx21*^–/–^ double-knockout mice^7^. In our study, we show that *Tbx21* is normally expressed at low levels in wild-type NK cells across the different mouse strains, but was downregulated in GFI1^–/–^ NK cells. While EOMES expression was restored to wild-type levels in GFI1^–/–^FOXO1^–/–^ NK cells, combined loss of GFI1 and FOXO1 did not rescue T-BET expression. Interestingly, chromatin accessibility at the *Tbx21* locus was rescued in GFI1^–/–^FOXO1^–/–^ NK cells, but this did not rescue T-BET protein expression, suggesting that GFI1 fine-tunes T-BET during NK cell differentiation in a FOXO1-independent manner. Furthermore, complete loss of T-BET in FOXO1^–/–^T-BET^–/–^ NK cells in vivo has previously failed to rescue mature NK cell development^7^. However, Deng et al. showed that mice carrying only a single copy of *Tbx21* substantially restored NK cells to wild-type levels^7^. Thus, consistent with previous findings, T-BET is essential for NK cell development^34,35,38^, but that the level of expression is regulated in vivo either directly or indirectly by FOXO1^7^. Together, these data indicate that GFI1 functions as a developmental checkpoint by repressing EOMES and FOXO1, thereby regulating the EOMES–T-BET balance essential for NK cell development.

Loss of FOXO1 alone resulted in an expansion of NK cell populations accompanied by increased EOMES expression. Although *Gfi1* mRNA levels were also elevated, GFI1 primarily functions through direct binding to *cis*-regulatory elements to mediate potent transcriptional repression and autoregulation^39^. In this context, increased transcript levels likely reflect amplification of a non-functional *Gfi1* allele rather than enhanced GFI1 activity.

Dual deletion of FOXO1 and GFI1 rescued many features of the GFI1-induced defects, partially restoring elements of the transcriptional network and surface activating and inhibitory molecules. While some integrins and homing molecules, such as *Cd226* and *Itgam,* were fully rescued, others, such as *Itga4* and *Itgax,* were not rescued. These findings indicate that FOXO1 and GFI1 converge on a shared core NK cell programme, while also independently regulating distinct and essential transcriptional modules. Further studies will be necessary to fully delineate these parallel regulatory pathways.

In summary, we identify GFI1 as a cell-intrinsic regulator of NK cell development that governs maturation and tissue distribution by modulating a core transcriptional programme centred on FOXO1. This programme enables peripheral immature NK cells to progress through discrete maturation stages and their effective distribution to tissues. Mechanistically, we show that GFI1-dependent defects are not rescued by EOMES deletion alone, but are largely mediated through FOXO1, which in turn coordinates EOMES and T-BET expression. Together, these findings define a negative regulatory axis that integrates a broader transcriptional network, which ensures the balance between EOMES and T-BET in NK cell development and provides a framework for how NK cell homoeostasis and protective function are maintained across diverse tissue environments.

## Methods

### Mice

*Gfi1*^*tdTomato/+*^ ^21,40^, *Gfi1*^*fl/fl*^ ^22^, *Foxo1*^*tm1Rdp*^ (*Foxo1*^*fl/fl*^)^41^*, Eomes*^*tm1Gtbz*^ (*Eomes*^*fl/fl*^)^42^ and *Ncr1*^*tm1.1(iCre)Viv*^(*Ncr*^*iCre*^) ^11^ mice have been described previously (Supplementary Data 7). B6.SJL-Ptprc^a^ Pepc/BoyJ (CD45.1^+/+^) and C57BL/6 mice were purchased from The Jackson Laboratory. *Gfi1*^*tdTomato/+*^ were backcrossed for at least 10 generations to the C57BL/6 background. All mice were used at 6–12 weeks old. Both male and female mice were used. Mice were bred and maintained under specific pathogen-free conditions at the animal facility of The University of Queensland or the WEHI. Mice were housed under a 12 h light/12 h dark cycle at 22 °C ± 2 °C and 55% ± 15% humidity. Euthanasia was performed using CO_2_ asphyxiation. All experiments were approved by the University of Queensland and Walter and Eliza Hall Institute Animal Ethics Committees and were carried out in accordance with the Guidelines of the Australian Code for the Care and Use of Animals of the National Health and Medical Research Council of Australia. All experimental procedures were approved by the Animal Ethics Committees of the respective institutions.

### Cell lines and virus infection

B16-F10 (CRL-6475) and M2-10B4 (CRL-1972) cells were obtained from ATCC. All cell lines were maintained in DMEM supplemented with 10% heat-inactivated foetal calf serum (FCS), 2 mM L-glutamine, 100 U/mL penicillin and 100 μg/mL streptomycin. MCMV is derived from pSM3fr-MCK-2fl clone 3.3 BAC^43^. MCMV was reconstituted by BAC transfection of M2-10B4 cells. The virus was then propagated on M2-10B4 cells. Virus stocks were generated as previously described^43,44^. For analysing NK cell responses, mice were infected with 2 × 10^5^ plaque-forming units (PFU) of MCMV intraperitoneally. To determine MCMV replication in vivo, mice were intraperitoneally infected with 1 × 10^6^ PFU of MCMV, and infected tissues were homogenised and titrated on M2-10B4 cells as previously described^45^.

### Cell isolation and flow cytometric analyses

Single cell suspensions were generated by forcing tissues through 70 μm cell strainers. Red blood cells (RBC) were lysed using ACK buffer (150 mM NH_4_Cl, 10 mM KHCO_3_, 0.1 mM EDTA). Blood samples were collected via retro-orbital bleeding, and RBC were lysed using ACK buffer. Lungs were perfused with approximately 5 mL phosphate-buffered saline (PBS) via the right ventricle to remove circulating blood. Then, lungs were placed in collagenase type IV (1 mg/mL, Worthington) deoxyribonuclease I (200 μg/mL, Roche) and dispase (0.4 U/mL; Gibco) in complete RPMI medium (RPMI 1640 medium containing 10% heat-inactivated FCS, 1 mM l-glutamine, 100 U/mL penicillin, 100 μg/mL streptomycin and 50 μM β-mercaptoethanol) and homogenized using the gentleMACS Dissociator (Miltenyi Biotec) mouse lung digestion protocol setting. After dissociation, mononuclear cells were purified by gradient centrifugation using a 40–80% Percoll gradient. Lymphocytes were isolated from the liver single cell suspension using a 33% isotonic Percoll gradient. Bone marrow cells were isolated from long bones by perfusion and filtered through 70 μm cell strainers. Cell suspensions were blocked with PBS containing 5 mg/mL anti-CD16/CD32 (2.4G2) and stained (30 min on ice) with fluorophore-conjugated antibodies or reagents in FACS buffer (PBS containing 2.5% heat-inactivated FCS and 50 mM EDTA), unless stated otherwise. For intracellular staining, surface-labelled cells were fixed using eBioscience Foxp3/Transcription Factor Staining Buffer (Thermo Fisher), then stained for intracellular cytokines or transcription factors. For cytokine detection, single cell suspensions were stimulated with phorbol 12-myristate 13-acetate (100 ng/mL), ionomycin (100 ng/mL) and brefeldin A (10 μg/mL) in complete RPMI 1640 medium for 4 h followed by surface and intracellular staining. Live cells were identified by exclusion staining with a fixable viability dye (BD Biosciences or BioLegend) or 7-AAD (BD Biosciences). All antibodies and staining reagents used in the study are outlined in Supplementary Data 8 and the reagents outlined in Supplementary Data 9. Flow cytometry analysis was performed on a Cytek Aurora (Cytek Biosciences) or LSRFortessa X-20 (BD Biosciences), and analysis undertaken using FlowJo analysis software v.10.10 (BD Biosciences).

### Generation of mixed bone marrow chimeras

CD45.1^+/+^ recipient mice (6–10 weeks old) were lethally irradiated with two doses of 5.5 Gy (3 h apart). Bone marrow cells were isolated from congenically-labelled *Ncr*^*iCreT/+*^ (CD45.1^+^CD45.2^+^, wildtype) and *Ncr*^*iCreT/+*^*Gfi1*^*fl/fl*^ (CD45.2^+/+^) donor mice by flushing the femoral and tibial bones with sterile FACS buffer to make a single cell suspension. RBCs were lysed using ACK buffer, then washed twice with FACS buffer. Live cells were enumerated using Trypan blue exclusion. *Ncr*^*iCreT/+*^ and *Ncr*^*iCreT/+*^*Gfi1*^*fl/fl*^ bone marrow cells were mixed 1:1, and 2-4 × 10^6^ mixed bone marrow cells were then adoptively transferred into the irradiated recipients. Chimeric mice were allowed 6–10 weeks to fully reconstitute their hemopoietic compartment with donor bone marrow cells before virus infection or tumour inoculation.

### Pulmonary metastasis

B16-F10 murine melanoma cells were maintained in DMEM supplemented with 10% heat-inactivated FCS, 2mM L-Glutamine, 100 U/mL penicillin, and 100 μg/mL streptomycin. 2 × 10^5^ B16-F10 cells were injected via the tail vein of recipient mice. Animals were monitored daily for clinical and behavioural signs to ensure welfare and determine humane endpoints, in accordance with protocols approved by the Animal Ethics Committees at the University of Queensland and the Walter and Eliza Hall Institute. After 14 days, the lungs, liver, long bones and kidneys were harvested, fixed in formalin, and B16-F10 tumour metastases were counted using a dissecting microscope.

### In vitro NK cell culture and killing assay

For in vitro NK cell culturing, splenocytes were isolated from naïve mice and NK cells were enriched using EasySep Mouse NK Isolation Kit (StemCell Technologies) or MACS NK Cell Isolation Kit, mouse (Miltenyi Biotec, Inc.) according to the manufacturer’s protocol and then labelled with CellTrace Violet (CTV) (Invitrogen). Immature (Imm, CD11b^-^KLRG1^-^), M1 (CD11b^+^KLRG1^-^) and M2 (CD11b^+^KLRG1^+^) NK cell subsets were sorted using BD FACSAria Fusion (BD Biosciences) or Aurora CS Cell Sorter (Cytek Biosciences). Sorted NK cells were cultured in Complete RPMI (RPMI 1640 medium containing 10% heat-inactivated FCS, 1 mM l-glutamine, 100 U/mL penicillin, 100 μg/mL streptomycin and 50 μM β-mercaptoethanol) with 50 ng/mL of IL-15 (PeproTech). Cells were analysed at day 0, 3 and 5 of culturing.

For the in vitro killing assay, NK cells were sorted and cultured in RPMI without phenol red (Gibco) supplemented with 10% heat-inactivated FCS, 1% 4-(2-hydroxyethyl)−1-piperazineethanesulfonic acid (HEPES), 1% GlutaMAX, 1% NEAA, 50 μM 2-mercaptoethanol, 1% sodium pyruvate and 50 ng/mL IL-15 for 6 days. B16-F10 target cells were labelled with 15 μM calcein AM (Invitrogen) in Complete RPMI without phenol red for 30 min at 37 °C. Target cells were washed twice after labelling, then cocultured with NK cells for 4 h at 37 °C. After 4 h of co-culture, Calcein AM release was quantified as relative fluorescence units (RFU) using CLARIOstar plus (BMG Labtech) fluorescence microplate reader and specific lysis was calculated with the following formula:

Specific lysis (%) = (ER [RFU] − SR [RFU])/(MR [RFU] − SR [RFU]) × 100; ER denotes experimental release, SR is spontaneous release, and MR is maximal release.

### RNA isolation and bulk RNA-seq

NK cell subsets were sorted from splenocytes, and total RNA was extracted using the RNeasy Plus Micro kit (Qiagen) according to the manufacturer’s instructions. The quality and integrity of RNA was measured using Bioanalyzer or TapeStation systems (Agilent Technologies). Libraries were prepared using TruSeq Stranded Total RNA kit (Illumina) and sequenced ( > 50 million reads/sample) using NovaSeq S1 PE100 flow cell (Illumina) or NovaSeq SP 100 flow cell (Illumina).

RNA-seq read quality was assessed with *fastp*^46^ to trim low-quality reads. Reads were mapped to the mouse genome (mm10) using STAR v2.7.10^47^, and mapped reads were quantified with *featureCounts* v2.0.1^48^. Read counts were normalised and differential gene expression quantified with DESeq2 v1.4.0. A log-fold change larger than one and a false discovery rate cut-off of 5% was used to select significantly over- and under-represented genes. Volcano plots and heatmaps were plotted using *EnhancedVolcano* v1.18.0 and *pheatmap* v1.0.12 packages, respectively.

### Single cell multiomic sequencing

Single-cell multiomic sequencing was performed using BD Rhapsody WTA + ATAC analyses kits (BD Biosciences). NK cells were sorted from the spleen of naïve mice, and cells were pooled from 3–5 mice to make a cell suspension (1 × 10^5^ cells) for nuclei isolation. NK cells were spun at 400 × *g* for 5 min. Then, the pellet was resuspended in 100 µL of chilled cell lysis buffer (10 mM Tris-HCl, 10 mM NaCl, 3 mM MgCl_2_, 0.1% tween-20, 0.1% IGEPAL CA-630, 0.01% digitonin, 1 mM dithiothreitol, 1 U/μL RNase inhibitor and 1% BSA) for 3 min to isolate nuclei. Cell lysis was stopped by adding 1 mL of wash buffer (10 mM Tris-HCl (pH 7.4), 10 mM NaCl, 3 mM MgCl_2_, 0.1% Tween 20, 1 mM dithiothreitol, 1 U/µL RNase inhibitor and 1% BSA). Nuclei were washed twice with wash buffer and then resuspended in chilled Nuclei Buffer (1 × Nuclei Buffer, 0.1 mM dithiothreitol and 1 U/µL RNase inhibitor). A total of 50,00 NK cell nuclei were incubated at 37 °C in tagmentation mix according to BD Rhapsody single-cell ATAC-seq and mRNA Whole Transcriptome Analysis library preparation protocol v23-24474(01). After 30 min incubation, nuclei were loaded onto the BD Rhapsody 8-Lane Cartridge followed by loading BD Rhapsody Enhanced Cell Capture Beads V3. Single-cell ATAC and single-cell RNA libraries were prepared as per the user guide. For the ATAC-seq, libraries were sequenced using a NextSeq 200 P4 flow cell (Illumina) with the following read protocol: 50 cycles (read 1), 8 cycles (index 1), 60 cycles (index 2) and 50 cycles (read 2). RNA libraries were sequenced on a NextSeq 200 P4 flow cell (Illumina) with the following cycle settings: 51 cycles (read 1), 8 cycles (i7 index read), 8 cycles (i5 index read) and 71 cycles (read 2).

### Single-cell multiomic data processing

Single-cell RNA-seq and single-cell ATAC-seq FASTQ files were pre-processed using BD Rhapsody Sequence Analysis Pipeline v1.2 to map RNA transcripts and ATAC peaks to the mm39 genome. Seurat objects were created using RNA transcript matrix files, and ATAC-seq data (ATAC-seq fragments file) was added using *CreateChromatinAssay*. Quality control was performed by filtering cells with the following criteria: between 200 and 2000 total RNA features, between 1000 and 20,000 total ATAC counts and percent mitochondrial counts < 20. Following quality control, the object was split into independent RNA-seq and ATAC-seq objects for individual processing.

RNA-seq expression count data for each dataset was normalised using *SCTransform*. SCTransformed data from multiple datasets (e.g., *Ncr*^*iCreT/+*^ and *Ncr*^*iCreT/+*^*Gfi1*^*fl/fl*^ NK cells) were prepared for merging and integration using *PrepSCTIntegration* and principal component analysis was performed to find 50 nearest neighbours for each dataset using the Louvain algorithm. After preprocessing, datasets were merged and integrated using the *IntegrateData* (RPCAIntegration) function. For ATAC-seq datasets, consensus peaks were identified using Signac *CallPeaks* function with MACS3 and non-standard chromosome peaks were removed. Term-frequency inverse-document-frequency was calculated, and singular value decomposition was run using Signac *RunSVD* function. Then, dimension reduction was performed using latent semantic indexing (LSI), followed by graph-based clustering on the LSI components 2 to 30. ATAC-seq datasets were integrated using integration anchors calculated using *FindIntegrationAnchors* and merged using *IntegrateEmbeddings*. The integrated RNA-seq data was used to generate a UMAP using a joint weighted nearest neighbour map and unsupervised clustering was performed. UMAP plots showing NK cell clusters were produced with the *DimPlot* function and UMAP based gene expression plots were produced with scCustomize package v3.2.0 using the Viridis colour scale. Differentially expressed genes (DEGs) and differentially accessible regions (DARs) between two groups of cells were calculated using the *FindMarkers* function. Chromatin track plots were generated using the Signac *CoveragePlot* function. Differential abundance of cells in neighbourhoods was quantified using MiloR v2.2.0^23^. Pathway enrichment analysis was performed using AUCell v1.28.0. Immature NK cell signature was defined by comparing CD11b^-^KLRG1^-^ and CD11b^+^KLRG1^+^ *Ncr*^*iCreT/+*^ NK cell subsets and quantifying differentially expressed genes. DEGs were ranked by Benjamini-Hochberg procedure (fdr) corrected *p-*values and the top 100 DEGs were used to calculate the signature score and applied to single-cell data using the *AddModuleScore* function. Mature NK cell signature was defined similarly by comparing CD11b^+^KLRG1^+^ *Ncr*^*iCreT/+*^ NK cell transcriptome to CD11b^-^KLRG1^-^ and CD11b^+^KLRG1^-^ *Ncr*^*iCreT/+*^ NK cells.

### Quantification and statistical analysis

Statistical analyses were performed using Prism 10 software (GraphPad). Bar graphs show the mean ± SEM. Statistical tests for each dataset are indicated in the figure legend; exact *n-*values and *p-*values are indicated. Statistical significance was considered when the *p-*value was < 0.05.

### Reporting summary

Further information on research design is available in the Nature Portfolio Reporting Summary linked to this article.

## Supplementary information

Supplementary Information

Description of Additional Supplementary Files

Supplementary Data 1

Supplementary Data 2

Supplementary Data 3

Supplementary Data 4

Supplementary Data 5

Supplementary Data 6

Supplementary Data 7

Supplementary Data 8

Supplementary Data 9

Reporting Summary

Transparent Peer Review file

## Source data

Source Data

## Data Availability

All data supporting the findings of this study are included in the main text, supplementary information and source data files. Raw data files for the bulk RNA sequencing have been deposited in the NCBI Gene Expression Omnibus (GEO) under accession number GSE278867. Single-cell multiomic-seq data can be accessed using the accession number GSE313719. Source data are provided in this paper.
