# Supplementary material for: The GFI1–FOXO1 axis regulates NK cell maturation and function
Source: Nat Commun. 2026 Apr 22;17:5593. doi: 10.1038/s41467-026-72022-6 (PMC13315798; doi:10.1038/s41467-026-72022-6)
Supplement: Supplementary file 1 — Supplementary Information [file 41467_2026_72022_MOESM1_ESM.pdf]

Supplementary Materials for

# GFI1-driven epigenetic programs regulate NK cell maturation and function

Qiutong Huang *et al*.

**The PDF file includes:**
Supplemental Figs. 1 to 11

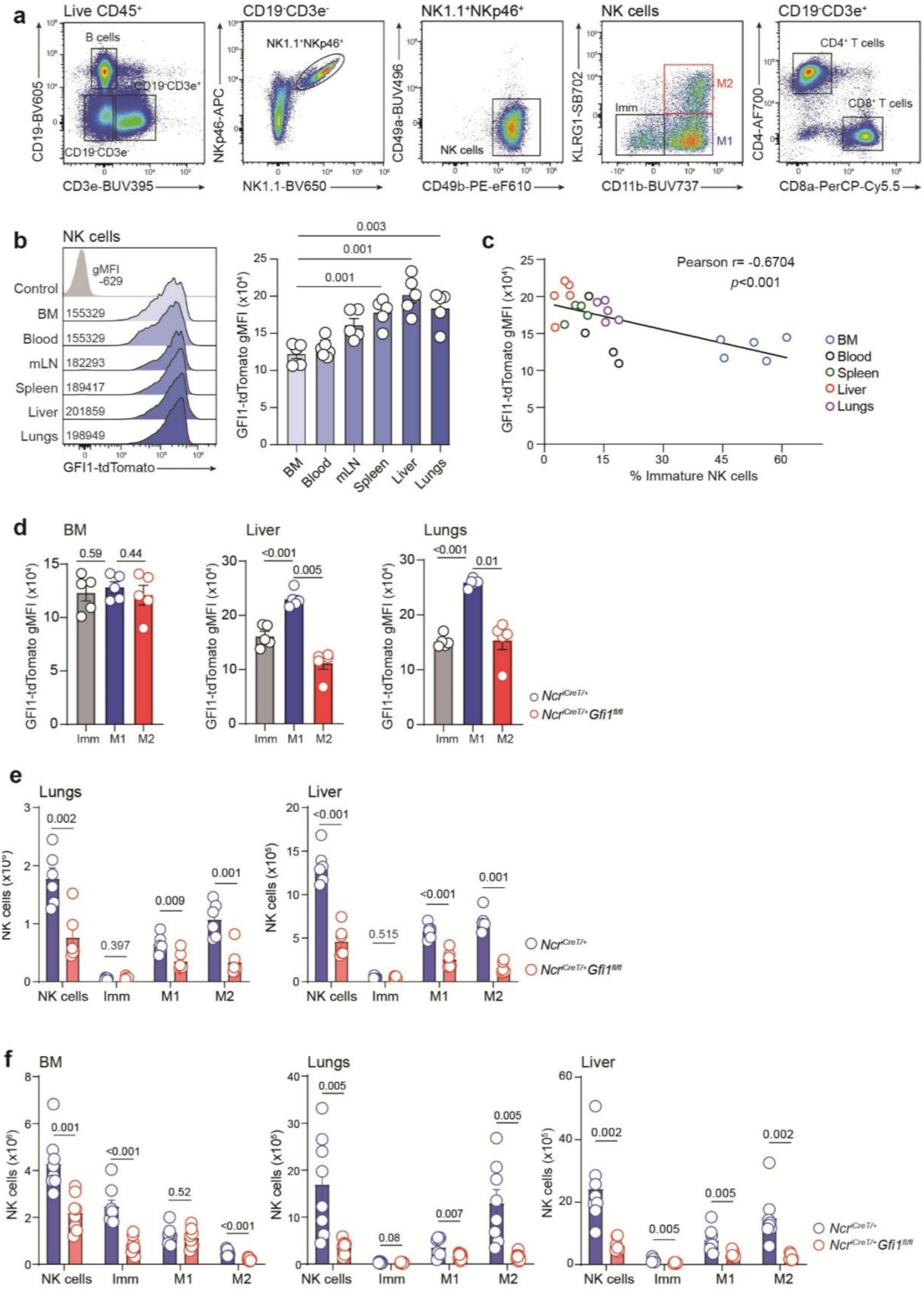
a
Live CD45+
B cells
CD19-CD3e+
CD19-CD3e-
CD19-BV605
CD3e-BUV395
CD19-CD3e-
NK1.1+NKp46+
NKp46-APC
NK1.1-BV650
NK1.1+NKp46+
CD49a-BUV496
NK cells
CD49b-PE-eF610
NK cells
KLRG1-SB702
Imm
M2
M1
CD11b-BUV737
CD19-CD3e+
CD4+ T cells
CD8+ T cells
CD4-AF700
CD8a-PerCP-Cy5.5
b
NK cells
gMFI
-629
Control
BM 155329
Blood 155329
mLN 182293
Spleen 189417
Liver 201859
Lungs 198949
GFI1-tdTomato
0.003
0.001
0.001
GFI1-tdTomato gMFI (x10⁴)
BM
Blood
mLN
Spleen
Liver
Lungs
c
Pearson r= -0.6704
p<0.001
GFI1-tdTomato gMFI (x10⁴)
% Immature NK cells
BM
Blood
Spleen
Liver
Lungs
d
BM
0.59
0.44
Liver
<0.001
0.005
Lungs
<0.001
0.01
GFI1-tdTomato gMFI (x10⁴)
Imm
M1
M2
NcriCreT/+
NcriCreT/+Gfi1fl/fl
e
Lungs
0.002
0.397
0.009
0.001
Liver
<0.001
0.515
<0.001
0.001
NK cells (x10⁵)
NK cells
Imm
M1
M2
NcriCreT/+
NcriCreT/+Gfi1fl/fl
f
BM
0.001
<0.001
0.52
<0.001
NK cells (x10⁶)
Lungs
0.005
0.08
0.007
0.005
NK cells (x10⁵)
Liver
0.002
0.005
0.005
0.002
NK cells (x10⁵)
NK cells
Imm
M1
M2
NcriCreT/+
NcriCreT/+Gfi1fl/fl

**Supplemental Figure 1. Genetic ablation of GFI1 impairs NK cell maturation. a**, Flow cytometric plots showing gating strategy for NK cell subsets isolated from spleen of *NCR*$^{iCreT/+}$ mice. **b**, Histogram (left) and geometric mean fluorescence intensity (gMFI) quantification (right) of GFI1-tdTomato in total NK cells (live $CD3^-CD3\varepsilon^-CD19^-NK1.1^+NKp46^+CD49b^+$) from different tissues of naive *Gfi1*$^{tdTomato/+}$ mice. Control shows NK cells from C57BL/6 spleen. Data show one of the two independent experiments as mean ± s.e.m. (*n*=5 biologically independent mice/ independent experiment). **c**, Correlation between GFI1-tdTomato gMFI of total NK cells and frequency of Imm ($CD11b^-KLRG1$) NK cells isolated from naïve *Gfi1*$^{tdTomato/+}$ mice tissue. Pearson correlation coefficient (r) and *p* value are shown. **d**, GFI1-tdTomato expression in Imm ($CD11b^-KLRG1^-$), M1 ($CD11b^+KLRG1^-$) and M2 ($CD11b^+KLRG1^+$) NK cells isolated from bone marrow (BM), liver and lungs of *Gfi1*$^{tdTomato/+}$ mice. Data show mean ± s.e.m. of one of two independent experiments (*n*=5 biologically independent mice/independent experiment). **e**, Total number of NK cell subsets among total NK cells isolated from the lungs and liver of *Ncr*$^{iCreT/+}$ and *Ncr*$^{iCreT/+}$*Gfi1*$^{fl/fl}$ mice. Data show mean ± s.e.m. of one of the three independent experiments (*n*=6 biologically independent mice/ independent experiment). **f**, Frequency of donor *Ncr*$^{iCreT/+}$ ($CD45.1^+CD45.2^+$) and *Ncr*$^{iCreT/+}$*Gfi1*$^{fl/fl}$ ($CD45.2^+$) NK cells isolated from different tissues 6 weeks after bone marrow reconstitution. Data pooled from two independent experiments is shown as mean ± s.e.m. (*n*=8 biologically independent mice). Statistical significance was calculated using one-way ANOVA and Tukey's post-test (**d**), a two-tailed Student's *t* test (**e**) or a two-tailed paired *t* test (**f**).

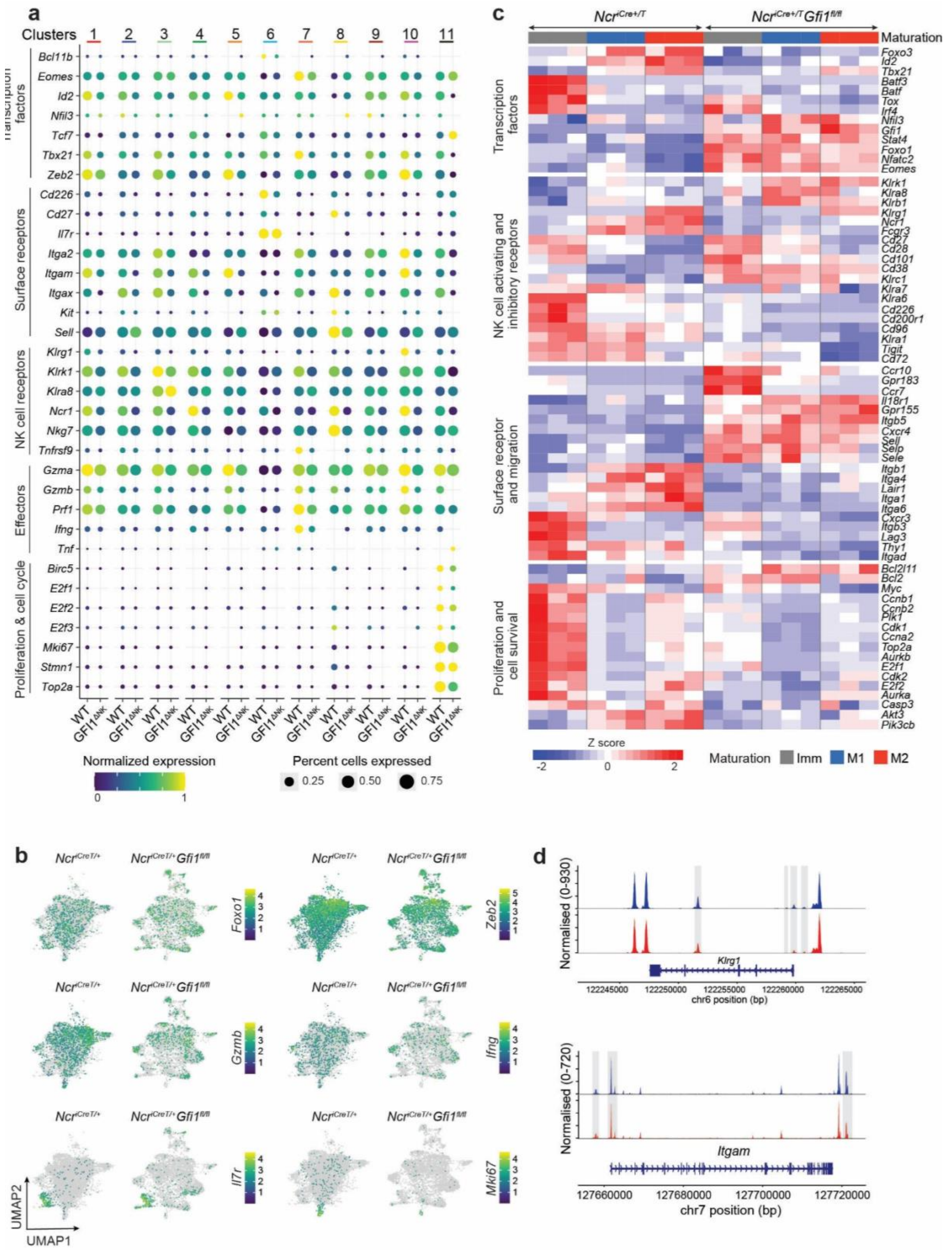
a
Clusters
Transcription factors
Surface receptors
NK cell receptors
Effectors
Proliferation & cell cycle
Normalized expression
Percent cells expressed
b
UMAP1
UMAP2
c
Maturation
Transcription factors
NK cell activating and inhibitory receptors
Surface receptor and migration
Proliferation and cell survival
Z score
Imm
M1
M2
d
Klrg1
chr6 position (bp)
Itgam
chr7 position (bp)

**Supplemental Figure 2. GFI1-mediated transcriptional regulation of NK cells.** **a**, Dot plot showing expression of selected genes in WT (*Ncr*$^{iCreT/+}$) and GFI1$^{\Delta NK}$ (*Ncr*$^{iCreT/+}$*Gfi1*$^{fl/fl}$) NK cells within different clusters calculated after RPCA integration of scRNA-seq data, with dot size indicating fraction of cells expressing gene and colour representing mean expression. **b**, UMAP showing normalized RNA expression of *Foxo1, Zeb2, Gzmb*, *Ifng*, *Il7r* and *Mki67* in NK cells as in **a**. **c**, Gene expression of selected differentially expressed genes ($p$<0.05) in Imm (CD11b$^-$KLRG1$^-$), M1 (CD11b$^+$KLRG1$^-$) and M2 (CD11b$^+$KLRG1$^+$) NK cells isolated from the spleen of naïve *Ncr*$^{iCreT/+}$ and *Ncr*$^{iCreT/+}$*Gfi1*$^{fl/fl}$ mice for bulk RNA-seq analysis. **d**, Histograms showing chromatin accessibility at *Klrg1* (top) and *Itgam* (bottom) loci in total *Ncr*$^{iCreT/+}$ (blue) and *Ncr*$^{iCreT/+}$*Gfi1*$^{fl/fl}$ (red) NK cells isolated as in **a**. Grey boxes highlight differentially accessible chromatin regions.

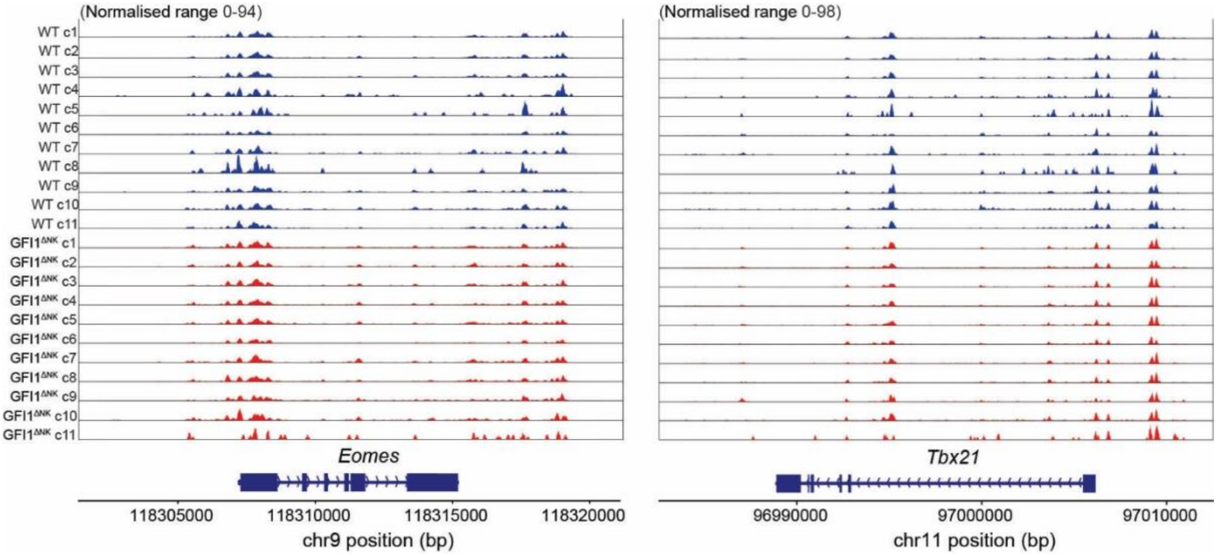


**Supplemental Figure 3. GFI1 epigenetically regulates NK cell transcription factors.** Histograms showing chromatin accessibility at *Eomes* (left) and *Tbx21* (right) loci in WT ($Ncr^{iCreT/+}$) and GFI1$^{\Delta NK}$ ($Ncr^{iCreT/+}Gfi1^{fl/fl}$) NK cells isolated from spleen for scMultiomic-seq and clustered using scRNA-seq data.

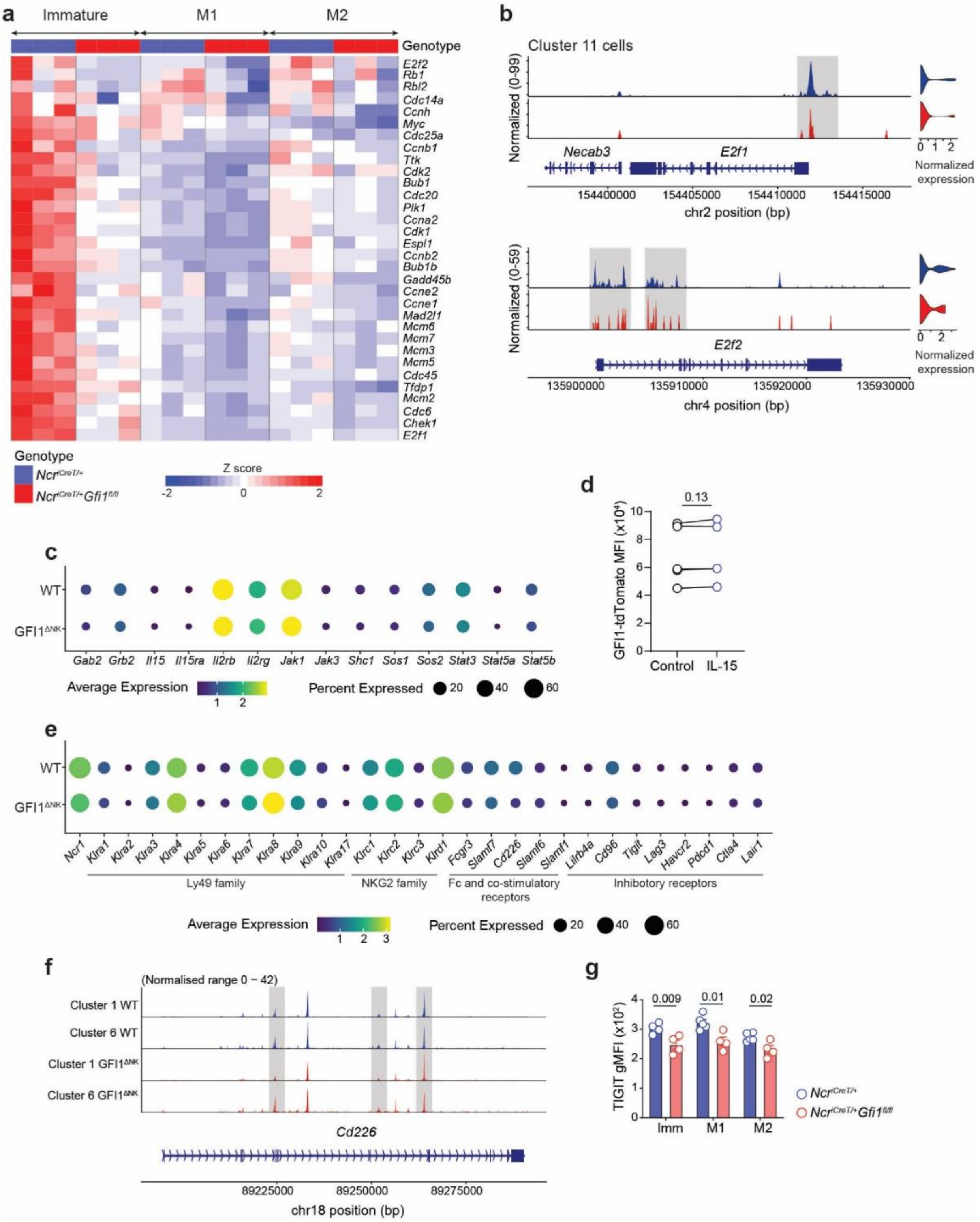
a
Immature
M1
M2
Genotype
E2f2
Rb1
Rbl2
Cdc14a
Ccnh
Myc
Cdc25a
Ccnb1
Ttk
Cdk2
Bub1
Cdc20
Plk1
Ccna2
Cdk1
Espl1
Ccnb2
Bub1b
Gadd45b
Ccne2
Ccne1
Mad2l1
Mcm6
Mcm7
Mcm3
Mcm5
Cdc45
Tfdp1
Mcm2
Cdc6
Chek1
E2f1
Z score
b
Cluster 11 cells
Normalized (0-99)
Necab3
E2f1
chr2 position (bp)
Normalized expression
Normalized (0-59)
E2f2
chr4 position (bp)
c
WT
Gab2
Grb2
Il15
Il15ra
Il2rb
Il2rg
Jak1
Jak3
Shc1
Sos1
Sos2
Stat3
Stat5a
Stat5b
Average Expression
Percent Expressed
d
GFI1-tdTomato MFI
Control
IL-15
e
WT
Ly49 family
NKG2 family
Fc and co-stimulatory receptors
Inhibitory receptors
Average Expression
Percent Expressed
f
(Normalised range 0 – 42)
Cluster 1 WT
Cluster 6 WT
Cd226
chr18 position (bp)
g
TIGIT gMFI
Imm
M1
M2

**Supplemental Figure 4. GFI1-mediated epigenetic control of NK cell proliferation and activatory receptors. a**, Gene expression of selected cell cycle related differentially expressed genes ($p$<0.05) in Imm ($CD11b^{-}KLRG1^{-}$), M1 ($CD11b^{+}KLRG1^{-}$) and M2 ($CD11b^{+}KLRG1^{+}$) NK cells isolated from the spleen of naïve $Ncr^{iCreT/+}$ and $Ncr^{iCreT/+}Gfi1^{fl/fl}$ mice for bulk RNA-seq analysis. **b**, Chromatin accessibility and gene expression of *E2f1* (top) and *E2f2* (bottom) loci in splenic NK cells within cluster 11, identified using unsupervised clustering based on scRNA-seq data. Grey boxes highlight differentially accessible chromatin regions. **c**, Dot plot showing expression of selected IL-15 signalling pathway genes in total WT ($Ncr^{iCreT/+}$) and $GFI1^{\Delta NK}$ ($Ncr^{iCreT/+}Gfi1^{fl/fl}$) NK cells isolated from spleen as in **b**. **d**, GFI1-tdTomato expression in splenic NK cells following 30 min *in vitro* culture with recombinant 50 ng/mL IL-15. Data from one of two independent experiments is shown ($n$=5 biologically independent mice/ independent experiment). **e**, Dot plot showing expression of selected NK cell activatory and inhibitory receptor genes in total WT ($Ncr^{iCreT/+}$) and $GFI1^{\Delta NK}$ ($Ncr^{iCreT/+}Gfi1^{fl/fl}$) NK cells isolated from spleen as in **b**, with dot size indicating fraction of cells expressing gene and colour representing mean expression. **f**, Chromatin accessibility at *Cd226* locus in different NK cell clusters, identified using unsupervised clustering based on scRNA-seq data. Grey boxes highlight differentially accessible chromatin regions. **g**, TIGIT expression in splenic NK cells isolated from $Ncr^{iCreT/+}$ and $NCR^{iCreT/+}Gfi1^{fl/fl}$ mice. Data show mean ± s.e.m. from one of two independent experiments ($n$=4 biologically independent mice/ genotype/ independent experiment). Statistical significance calculated using a two-tailed Student's $t$ test.

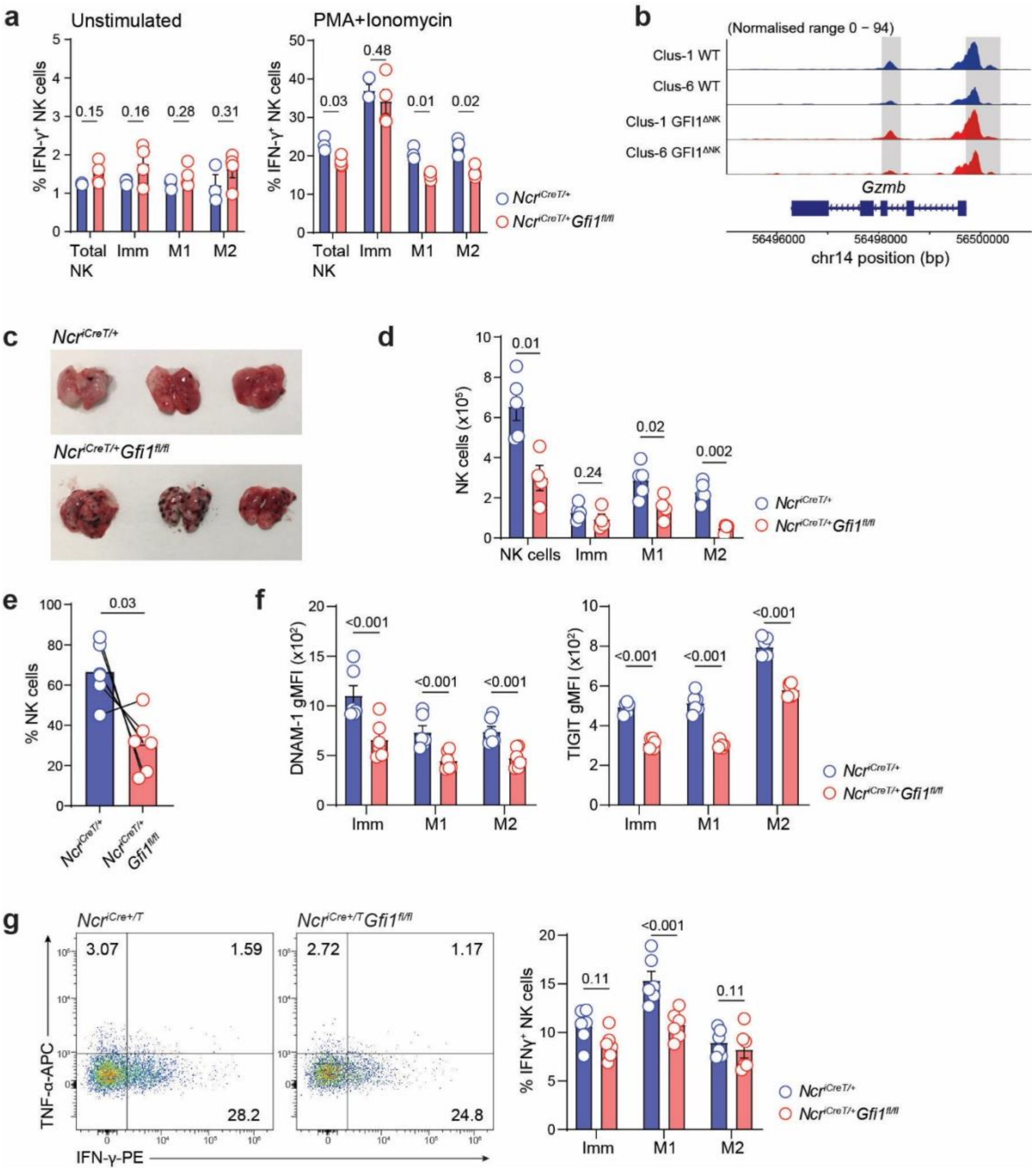
a
Unstimulated
PMA+Ionomycin
% IFN-γ+ NK cells
Total NK
Imm
M1
M2
b
(Normalised range 0 − 94)
Clus-1 WT
Clus-6 WT
Clus-1 GFI1ΔNK
Clus-6 GFI1ΔNK
Gzmb
chr14 position (bp)
c
NcriCreT/+
NcriCreT/+Gfi1fl/fl
d
NK cells (x10^5)
e
% NK cells
f
DNAM-1 gMFI (x10^2)
TIGIT gMFI (x10^2)
g
TNF-α-APC
IFN-γ-PE
% IFNγ+ NK cells

**Supplemental Figure 5. GFI1 is required for NK cell responses following tumour inoculation.** **a**, Frequency of IFN-ɣ$^+$ NK cells isolated from the spleen of *Ncr*$^{iCreT/+}$ and *Ncr*$^{iCreT/+}$*Gfi1*$^{fl/fl}$ mice quantified 4-hour after PMA+ionomycin stimulation *in vitro* in the presence of brefeldin A. Data from one of two independent experiments is shown as mean ± s.e.m. (*n*=3 biologically independent mice/independent experiment). **b**, Chromatin accessibility at *Gzmb* locus in different WT (*Ncr*$^{iCreT/+}$) and GFI1$^{\Delta NK}$ (*Ncr*$^{iCreT/+}$*Gfi1*$^{fl/fl}$) clusters, identified using unsupervised clustering based on scRNA-seq data. Grey boxes highlight differentially accessible chromatin regions. **c**, Infiltration of B16-F10 tumour cells in the lungs of *Ncr*$^{iCreT/+}$and *Ncr*$^{iCreT/+}$*Gfi1*$^{fl/fl}$ mice 14 days after intravenous inoculation of B16-F10 cells. Representative images show tumour metastases. Data representative of three independent experiments (*n*=17 or 19 biologically independent mice/genotype). **d**, Frequency of NK cell subsets isolated from spleen of tumour bearing *NCR*$^{iCreT/+}$ and *NCR*$^{iCreT/+}$*Gfi1*$^{fl/fl}$ mice at 14 days after B16-F10 cell injection. Data show mean ± s.e.m. (right) of one of two independent experiments (*n*=4 or 5 biologically independent mice/genotype/ independent experiment). Statistical significance was calculated using a two-tailed Student's *t* test. **e**, Frequency of *Ncr*$^{iCreT/+}$ (CD45.1$^+$CD45.2$^+$) and *Ncr*$^{iCreT/+}$*Gfi1*$^{fl/fl}$ (CD45.2$^{+/+}$) NK cells among total splenic NK cells at 14 days after tumour inoculation into chimeric mice (CD45.1$^+$) that received bone marrow cells 6 weeks prior to tumour inoculation. **f**, DNAM-1 and TIGIT expression in splenic NK cells as in **e**. **g**, Flow cytometric plots showing frequency of IFN-γ$^+$ and TNF-α$^+$ cells among lung *Ncr*$^{iCreT/+}$ and *Ncr*$^{iCreT/+}$*Gfi1*$^{fl/fl}$ NK cells as in **e** (left). Bar plots showing frequency of IFN-γ$^+$ cells among NK cells isolated from lungs of chimeric mice following 4-hour PMA+ionomycin stimulation (right). **e-g,** Data pooled from two independent experiments (*n*=6 biologically independent mice) show mean ± s.e.m. Statistical significance was calculated using a two-tailed Student's *t* test (A and C) or a two-tailed paired *t* test (**d-f**).

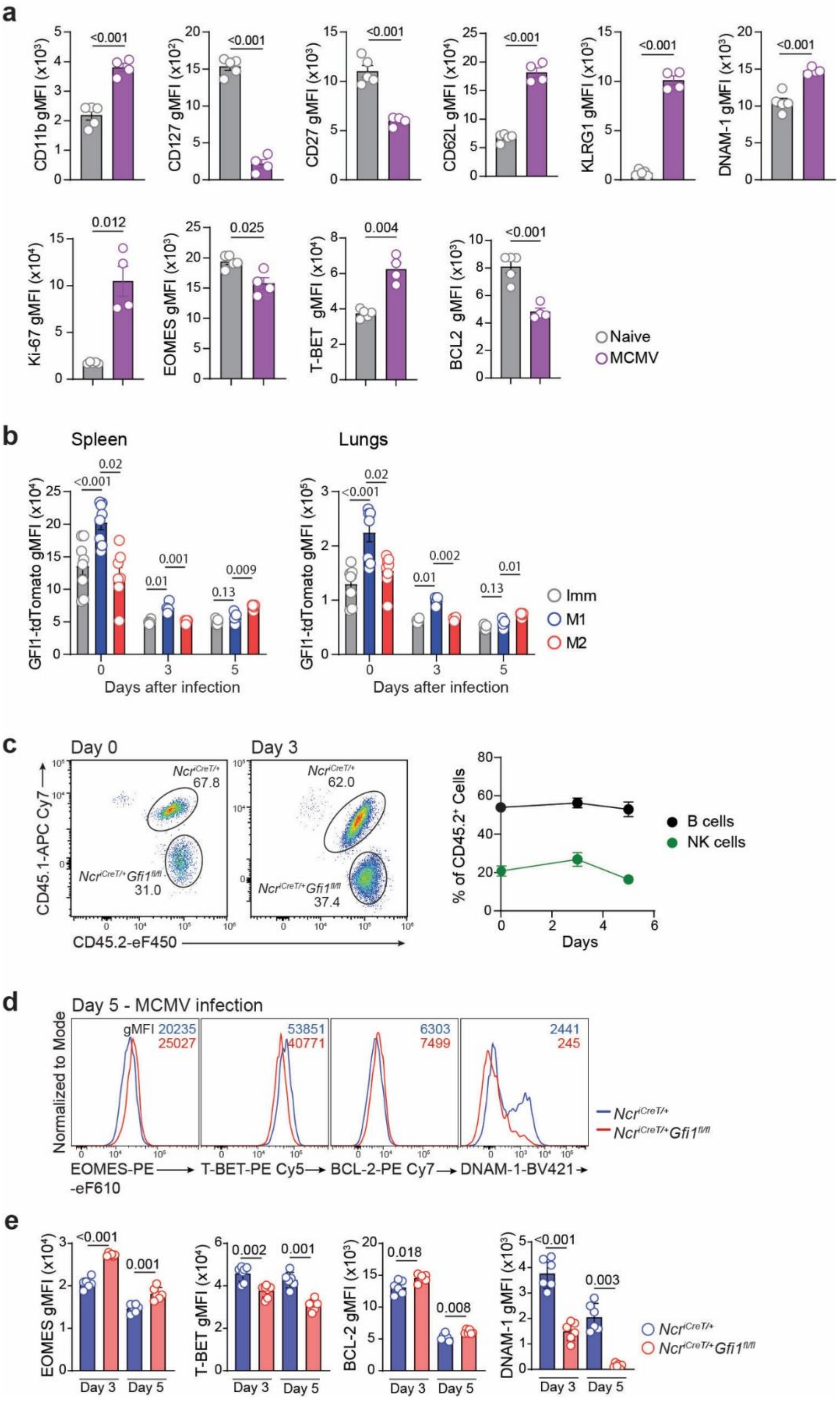
a
CD11b gMFI (x10³)
CD127 gMFI (x10²)
CD27 gMFI (x10³)
CD62L gMFI (x10⁴)
KLRG1 gMFI (x10³)
DNAM-1 gMFI (x10³)
Ki-67 gMFI (x10⁴)
EOMES gMFI (x10³)
T-BET gMFI (x10⁴)
BCL2 gMFI (x10³)
Naive
MCMV
b
Spleen
Lungs
GFI1-tdTomato gMFI (x10⁴)
GFI1-tdTomato gMFI (x10⁵)
Days after infection
Imm
M1
M2
c
Day 0
Day 3
CD45.1-APC Cy7
CD45.2-eF450
% of CD45.2⁺ Cells
Days
B cells
NK cells
d
Day 5 - MCMV infection
Normalized to Mode
gMFI
EOMES-PE -eF610
T-BET-PE Cy5
BCL-2-PE Cy7
DNAM-1-BV421
e
EOMES gMFI (x10⁴)
T-BET gMFI (x10⁴)
BCL-2 gMFI (x10³)
DNAM-1 gMFI (x10³)
Day 3
Day 5

**Supplemental Figure 6. GFI1 expression is required for NK cell proliferation and activation following virus infection.** **a**, Expression of CD11b, CD127, CD27, CD62L, KLRG1, DNAM-1, Ki-67, EOMES, T-BET and BCL-2 in total NK cells isolated from the spleen of naïve and infected $Gfi1^{dTomato/+}$ mice at 3 days after MCMV infection with $2\times10^5$ PFU inoculated intraperitoneally. Data show one experiment as mean ± s.e.m. (*n*=4 or 5 biologically independent mice/treatment group. **b**, GFI1-tdTomato expression in NK cell subsets isolated from the spleen and lungs as in A. Data pooled from two independent experiments shown as mean ± s.e.m. (day 0, *n*=8 biologically independent mice), or a single experiment (day 3, 5, *n*=4 or 5 biologically independent mice). **c**, Representative plots (left) and quantification (right) of $Ncr^{iCreT/+}$ and $Ncr^{iCreT/+}Gfi1^{fl/fl}$ NK cell frequency among total NK cells in peripheral blood at day 0 and 3 after MCMV infection of bone marrow chimeric mice reconstituted with $Ncr^{iCreT/+}$ and $Ncr^{iCreT/+}Gfi1^{fl/fl}$ bone marrow 6 weeks prior to virus infection. Data pooled from two independent experiments (*n*=7 biologically independent mice). **d**, EOMES, T-BET, BCL-2 and DNAM-1 expression in $Ncr^{iCreT/+}$ and $Ncr^{iCreT/+}Gfi1^{fl/fl}$ NK cells isolated from spleen of BM chimeric mice 5 days after infection as in **c**. Data is representative of two independent experiments (*n*=7 biologically independent mice/ independent experiment). **e**, EOMES, T-BET, BCL-2 and DNAM-1 expression in $Ncr^{iCreT/+}$ and $Ncr^{iCreT/+}Gfi1^{fl/fl}$ NK cells isolated from lungs of chimeric mice as in **c**. Data show mean ± s.e.m. pooled from two independent experiments (*n*=7 biologically independent mice). Statistical significance was calculate using a two-tailed Student's *t* test (**a**), two-way ANOVA with Tukey's post-test (**b**) or a two-tailed paired *t* test (**c**-**e**).

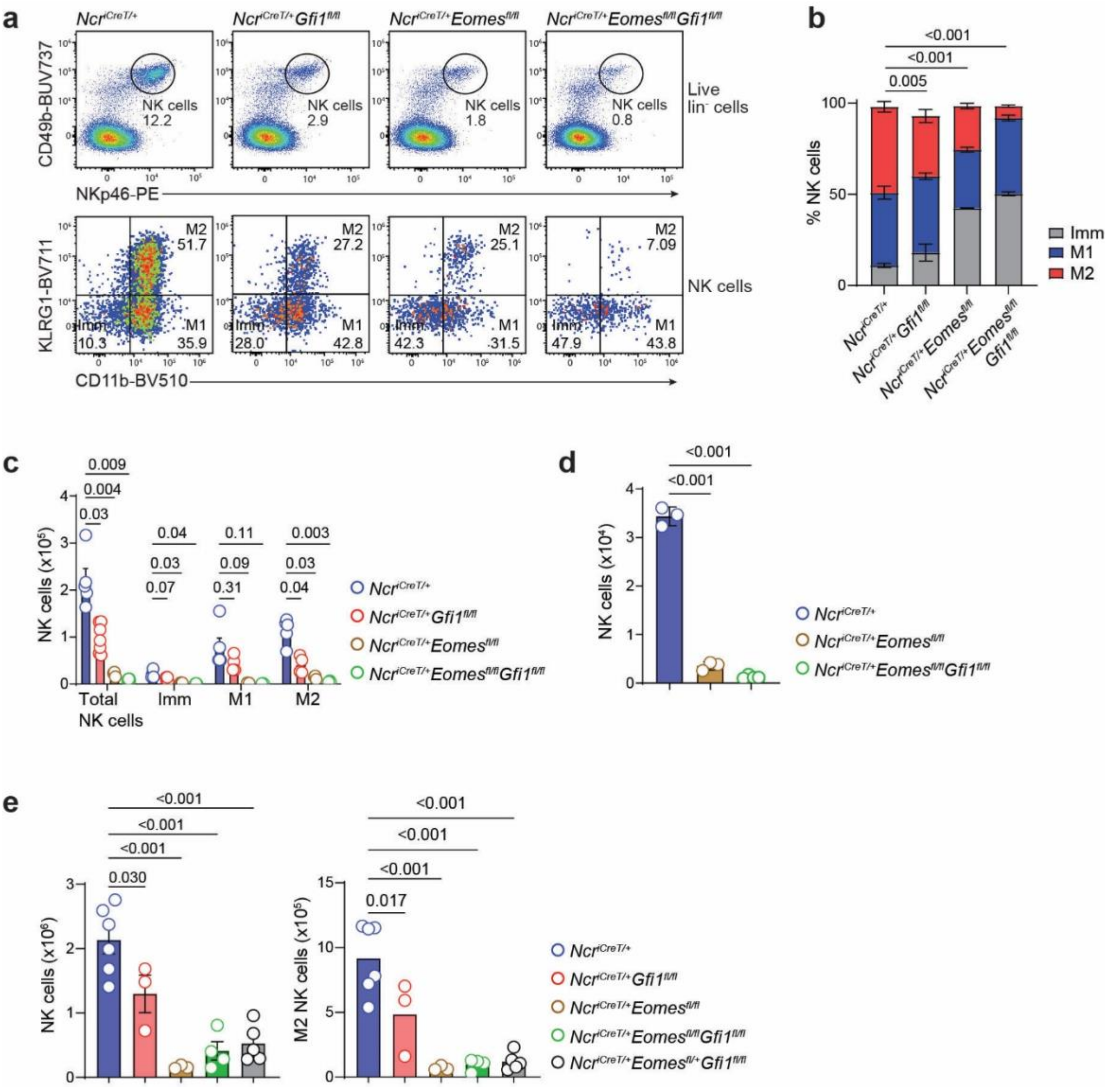
a
NcriCreT/+
NcriCreT/+Gfi1fl/fl
NcriCreT/+Eomesfl/fl
NcriCreT/+Eomesfl/flGfi1fl/fl
CD49b-BUV737
NKp46-PE
NK cells 12.2
NK cells 2.9
NK cells 1.8
NK cells 0.8
Live lin- cells
KLRG1-BV711
CD11b-BV510
NK cells
b
% NK cells
Imm
M1
M2
c
NK cells (x10⁵)
Total NK cells
Imm
M1
M2
d
NK cells (x10⁴)
e
NK cells (x10⁶)
M2 NK cells (x10⁵)
NcriCreT/+Eomesfl/+Gfi1fl/fl

**Supplemental Figure 7. Impact of genetic ablation of EOMES on NK cells.** **a**, Flow cytometry plots showing frequency of NK cells among lin$^-$ (live CD45$^+$CD3$^-$TCRβ$^-$CD19$^-$) cells isolated from spleen of *Ncr*$^{iCreT/+}$, *Ncr*$^{iCreT/+}$*Gfi1*$^{fl/fl}$, *Ncr*$^{iCreT/+}$*Eomes*$^{fl/fl}$ *and Ncr*$^{iCreT/+}$*Eomes*$^{fl/fl}$*Gfi1*$^{fl/fl}$ mice (top). NK cell subset frequency is shown among total NK cells (live CD45$^+$CD3$^-$TCRβ$^-$CD19$^-$NK1.1$^+$NKp46$^+$CD49b$^+$) isolated from spleen (bottom). **b**, Frequency of NK cell subsets isolated from spleen. **c**, Total number of NK cell subsets isolated from spleen of *Ncr*$^{iCreT/+}$, *Ncr*$^{iCreT/+}$*Gfi1*$^{fl/fl}$, *Ncr*$^{iCreT/+}$*Eomes*$^{fl/fl}$ *and Ncr*$^{iCreT/+}$*Eomes*$^{fl/fl}$*Gfi1*$^{fl/fl}$ mice. **d**, Total number of NK cells isolated from bone marrow of *Ncr*$^{iCreT/+}$, *Ncr*$^{iCreT/+}$*Eomes*$^{fl/fl}$ *and Ncr*$^{iCreT/+}$*Eomes*$^{fl/fl}$*Gfi1*$^{fl/fl}$ mice. **b**-**d**, Data from one experiment shown as mean ± s.e.m. (*n*= 3 or 4 biologically independent mice/genotype). **e**, Total number of NK cells isolated from spleen of *Ncr*$^{iCreT/+}$, *Ncr*$^{iCreT/+}$*Gfi1*$^{fl/fl}$, *Ncr*$^{iCreT/+}$*Eomes*$^{fl/fl}$, *Ncr*$^{iCreT/+}$*Eomes*$^{fl/fl}$*Gfi1*$^{fl/fl}$ *and Ncr*$^{iCreT/+}$*Eomes*$^{fl/+}$*Gfi1*$^{fl/fl}$ mice. Data pooled from two independent experiment shown as mean ± s.e.m. (*n*= 3, 4, 5 or 6 biologically independent mice/ genotype). Statistical significance was calculated using a one-way ANOVA and Dunnet's post-test (**b**, **d**) or a two-way ANOVA with Dunnet's post-test **(c**, **e)**.

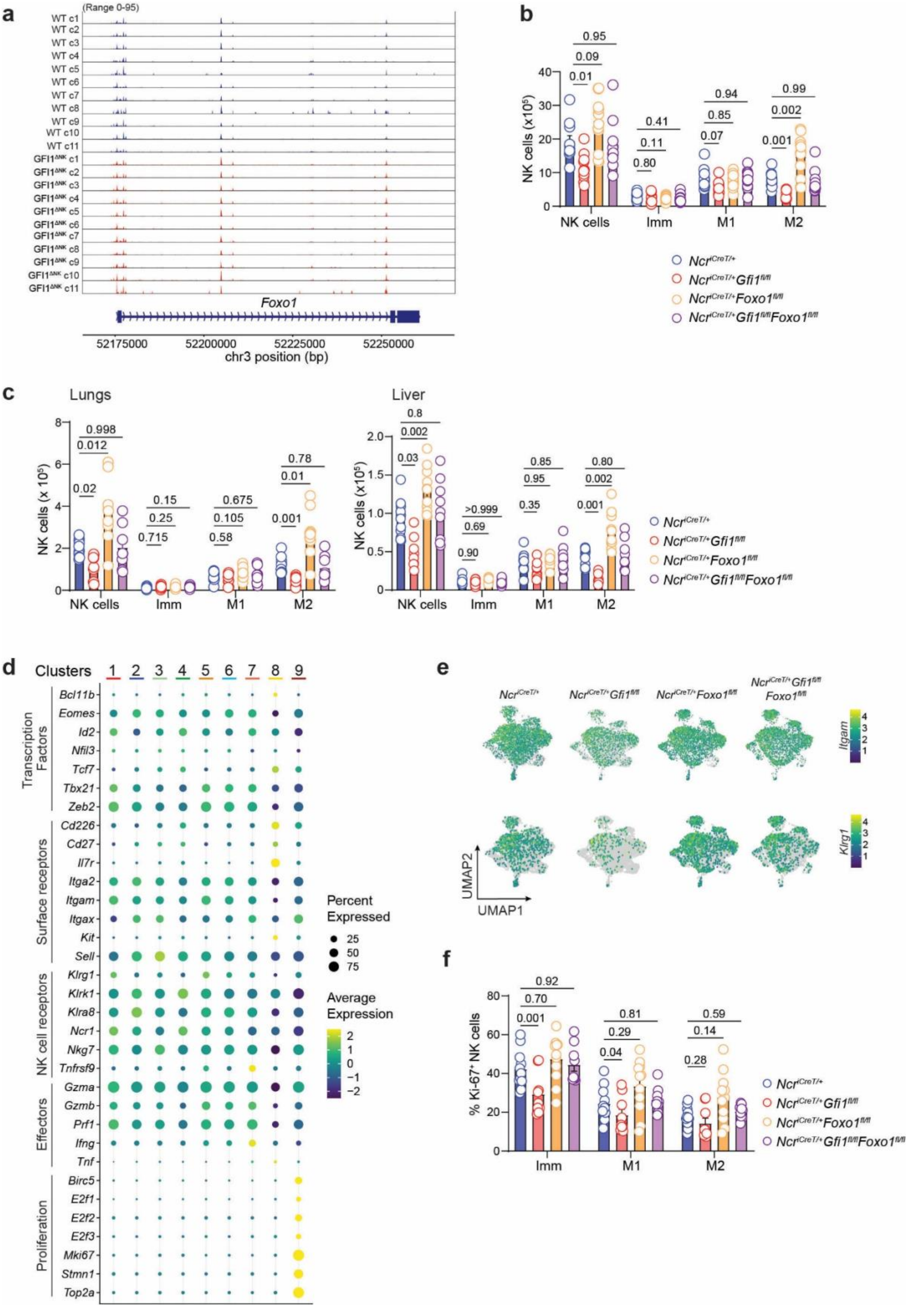
a
(Range 0-95)
Foxo1
chr3 position (bp)
b
NK cells (x10⁵)
NK cells
Imm
M1
M2
c
Lungs
Liver
d
Clusters
Transcription Factors
Surface receptors
NK cell receptors
Effectors
Proliferation
Percent Expressed
Average Expression
e
UMAP1
UMAP2
Itgam
Klrg1
f
% Ki-67⁺ NK cells

**Supplemental Figure 8. GFI1 epigenetically represses FOXO1 to regulate NK cell maturation. a**, Histograms showing normalised chromatin accessibility at *Foxo1* loci in WT (*Ncr*$^{iCreT/+}$) and GFI1$^{\Delta NK}$ (*Ncr*$^{iCreT/+}$*Gfi1*$^{fl/fl}$) NK cells isolated from the spleen for scMultiomic-seq and clustered using scRNA-seq data. **b-c**, Total number of NK cell and subsets isolated from the spleen (**b**), lungs and liver (**c**) of naïve *Ncr*$^{iCreT/+}$*, Ncr*$^{iCreT/+}$*Gfi1*$^{fl/fl}$*, Ncr*$^{iCreT/+}$*Foxo1*$^{fl/fl}$ *and Ncr*$^{iCreT/+}$*Gfi1*$^{fl/fl}$*Foxo1*$^{fl/fl}$ mice. Data pooled from two independent experiments shown as mean ± s.e.m. (*n*=10 or 13 biologically independent mice/ genotype). **d**, Dot plots showing average expression of selected NK cell genes within different clusters calculated after RPCA integration of *Ncr*$^{iCreT/+}$ (*n*=6225 cells), *Ncr*$^{iCreT/+}$*Gfi1*$^{fl/fl}$ (*n*=5654 cells), *Ncr*$^{iCreT/+}$*Foxo1*$^{fl/fl}$ (*n*=4909) and *Ncr*$^{iCreT/+}$*Gfi1*$^{fl/fl}$*Foxo1*$^{fl/f1}$ (*n*=4839) NK cells isolated from naïve mice, with dot size indicating fraction of cells expressing gene and colour representing mean expression. **e**, UMAP showing normalised *Itgam* (top) and *Klrg1* (bottom) expression in NK cells as in **d**. **f,** Frequency of Ki-67$^{+}$ NK cell subsets isolated from spleen of naïve *Ncr*$^{iCreT/+}$*, Ncr*$^{iCreT/+}$*Gfi1*$^{fl/fl}$*, Ncr*$^{iCreT/+}$*Foxo1*$^{fl/fl}$ *and Ncr*$^{iCreT/+}$*Gfi1*$^{fl/fl}$*Foxo1*$^{fl/fl}$ mice. Data pooled from two independent experiments shown as mean ± s.e.m. (*n*=10 or 13 biologically independent mice/ genotype). Statistical significance was calculated using a two-way ANOVA with Dunnett's post-test **(b, c, f)**.

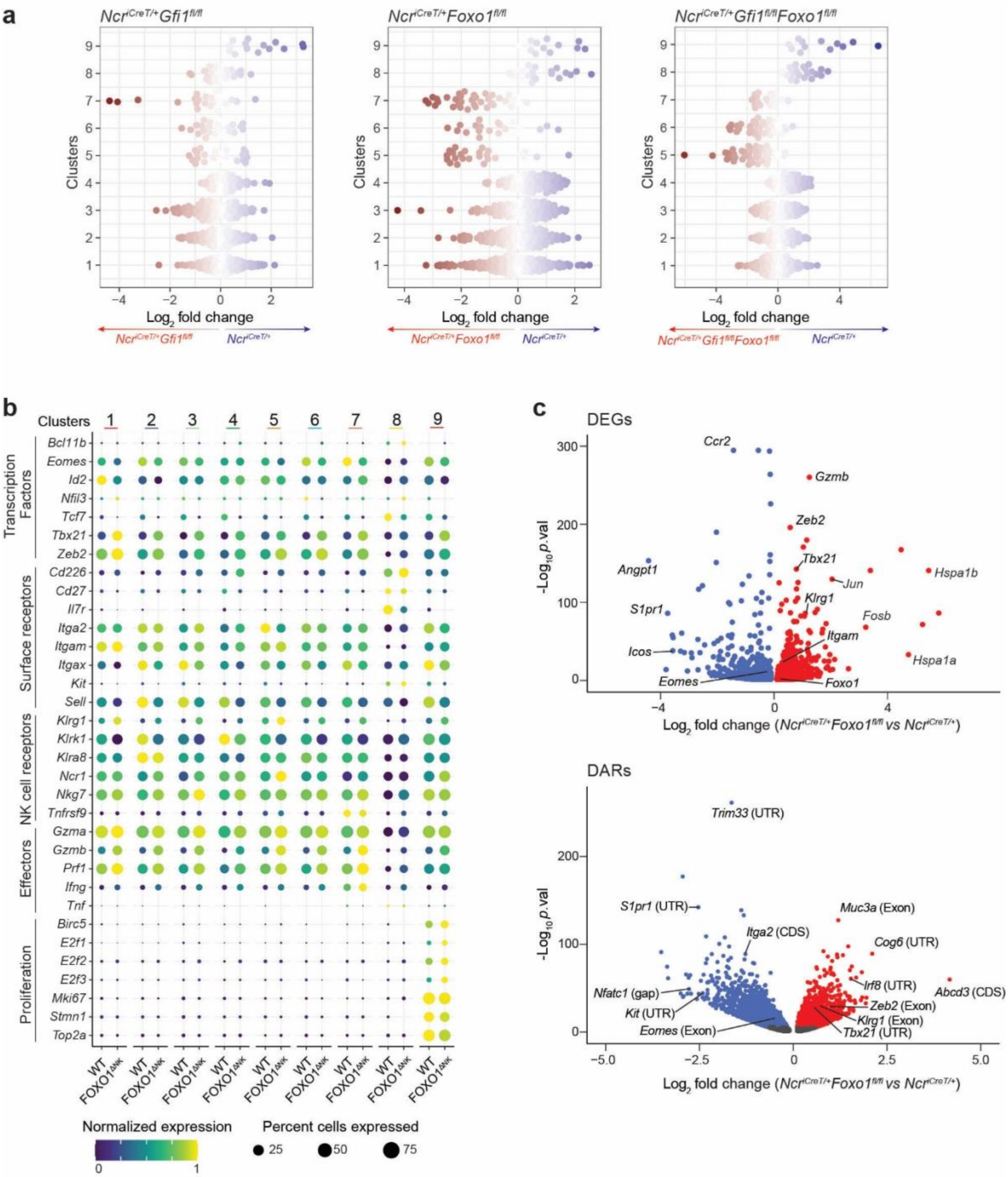
a
NcriCreT/+Gfi1fl/fl
NcriCreT/+Foxo1fl/fl
NcriCreT/+Gfi1fl/flFoxo1fl/fl
Clusters
Log2 fold change
NcriCreT/+
b
Clusters
Transcription Factors
Surface receptors
NK cell receptors
Effectors
Proliferation
Bcl11b
Eomes
Id2
Nfil3
Tcf7
Tbx21
Zeb2
Cd226
Cd27
Il7r
Itga2
Itgam
Itgax
Kit
Sell
Klrg1
Klrk1
Klra8
Ncr1
Nkg7
Tnfrsf9
Gzma
Gzmb
Prf1
Ifng
Tnf
Birc5
E2f1
E2f2
E2f3
Mki67
Stmn1
Top2a
WT
FOXO1ΔNK
Normalized expression
Percent cells expressed
c
DEGs
-Log10 p.val
Ccr2
Gzmb
Zeb2
Tbx21
Jun
Hspa1b
Angpt1
Klrg1
S1pr1
Fosb
Itgam
Icos
Hspa1a
Eomes
Foxo1
Log2 fold change (NcriCreT/+Foxo1fl/fl vs NcriCreT/+)
DARs
Trim33 (UTR)
S1pr1 (UTR)
Muc3a (Exon)
Itga2 (CDS)
Cog6 (UTR)
Irf8 (UTR)
Abcd3 (CDS)
Nfatc1 (gap)
Zeb2 (Exon)
Kit (UTR)
Klrg1 (Exon)
Eomes (Exon)
Tbx21 (UTR)

**Supplemental Figure 9. FOXO1-mediated transcriptional regulation of NK cells. a**, Differential neighbourhood abundance ($Log_2FC$), calculated using scRNA-seq data, is shown on the beeswarm plots. Blue dots indicate neighbourhoods significant enriched ($p$<0.05) for *Ncr*$^{iCreT/+}$, while red dots indicate neighbourhoods enriched for *Ncr*$^{iCreT/+}$*Gfi1*$^{fl/fl}$ (left), *Ncr*$^{iCreT/+}$*Foxo1*$^{fl/fl}$ (middle) or *Ncr*$^{iCreT/+}$*Gfi1*$^{fl/f}$*Foxo1*$^{fl/fl}$ (right) NK cells. Clusters were calculated after RPCA integration of NK cell scRNA-seq data. **B**, Dot plot showing expression of selected genes in WT (*Ncr*$^{iCreT/+}$) and FOXO1$^{\Delta NK}$ (*Ncr*$^{iCreT/+}$*Foxo1*$^{fl/fl}$) NK cells within different clusters calculated after RPCA integration of scRNA-seq data, with dot size indicating fraction of cells expressing gene and colour representing mean expression. **c**, Differentially expressed genes (top) and differentially accessible regions (bottom) in total NK cells detected using single-cell RNA-seq and single-cell ATAC-seq pseudobulk analysis, respectively. Blue dots indicate genes (DEGs) or regions (DARs) significantly upregulated in *Ncr*$^{iCreT/+}$ NK cells, while red dots indicate those significantly upregulated in *Ncr*$^{iCreT/+}$*Foxo1*$^{fl/fl}$ NK cells.

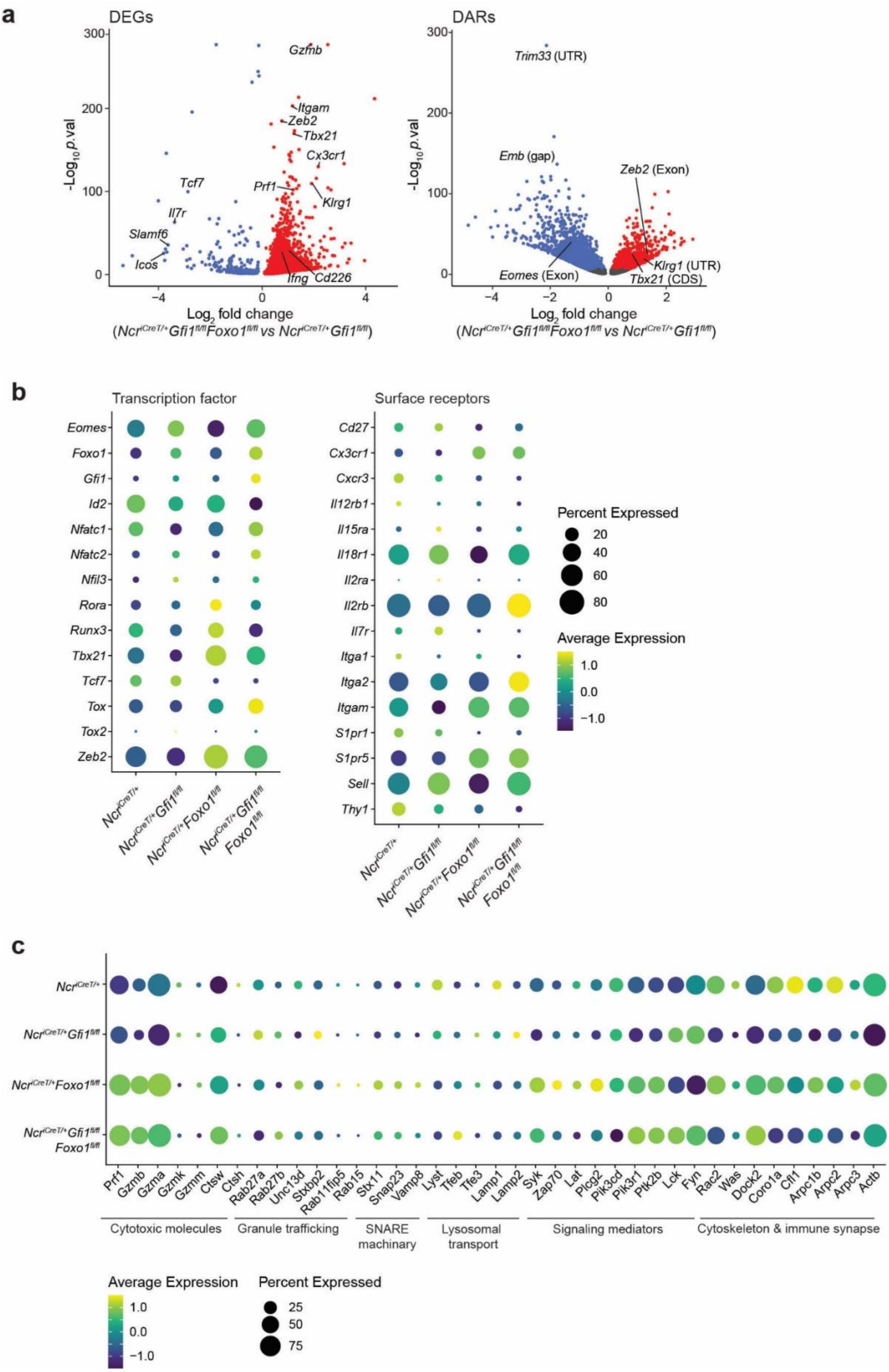
a
DEGs
DARs
Gzmb
Itgam
Zeb2
Tbx21
Cx3cr1
Prf1
Klrg1
Tcf7
Il7r
Slamf6
Icos
Ifng
Cd226
Trim33 (UTR)
Emb (gap)
Zeb2 (Exon)
Eomes (Exon)
Klrg1 (UTR)
Tbx21 (CDS)
-Log10 p.val
Log2 fold change
(NcriCreT/+Gfi1fl/flFoxo1fl/fl vs NcriCreT/+Gfi1fl/fl)
b
Transcription factor
Surface receptors
Eomes
Foxo1
Gfi1
Id2
Nfatc1
Nfatc2
Nfil3
Rora
Runx3
Tbx21
Tcf7
Tox
Tox2
Zeb2
Cd27
Cx3cr1
Cxcr3
Il12rb1
Il15ra
Il18r1
Il2ra
Il2rb
Il7r
Itga1
Itga2
Itgam
S1pr1
S1pr5
Sell
Thy1
NcriCreT/+
NcriCreT/+Gfi1fl/fl
NcriCreT/+Foxo1fl/fl
NcriCreT/+Gfi1fl/fl Foxo1fl/fl
Percent Expressed
20
40
60
80
Average Expression
1.0
0.0
−1.0
c
Prf1
Gzmb
Gzma
Gzmk
Gzmm
Ctsw
Ctsh
Rab27a
Rab27b
Unc13d
Stxbp2
Rab11fip5
Rab15
Stx11
Snap23
Vamp8
Lyst
Tfeb
Tfe3
Lamp1
Lamp2
Syk
Zap70
Lat
Plcg2
Pik3cd
Pik3r1
Ptk2b
Lck
Fyn
Rac2
Was
Dock2
Coro1a
Cfl1
Arpc1b
Arpc2
Arpc3
Actb
Cytotoxic molecules
Granule trafficking
SNARE machinary
Lysosomal transport
Signaling mediators
Cytoskeleton & immune synapse
Average Expression
1.0
0.0
−1.0
Percent Expressed
25
50
75

**Supplemental Figure 10. Genetic abrogation of FOXO1 partially restores NK cell differentiation.** **a**, Differentially expressed genes (right) and differentially accessible regions (left) in total NK cells detected using single-cell RNA-seq and single-cell ATAC-seq pseudobulk analysis, respectively. Blue dots indicate genes (DEGs) or regions (DARs) significantly upregulated in $Ncr^{iCreT/+}Gfi1^{fl/fl}Foxo1^{fl/fl}$ NK cells, while red dots indicate those significantly upregulated in $Ncr^{iCreT/+}Gfi1^{fl/fl}$ NK cells. **b**, Dot plot showing expression of selected transcription factor (left) and surface receptor (right) genes in total splenic NK cells, with dot size indicating fraction of cells expressing gene and colour representing mean expression. **c**, Dot plot showing expression of selected genes involved in degranulation in total splenic NK cells, with dot size indicating fraction of cells expressing gene and colour representing mean expression.

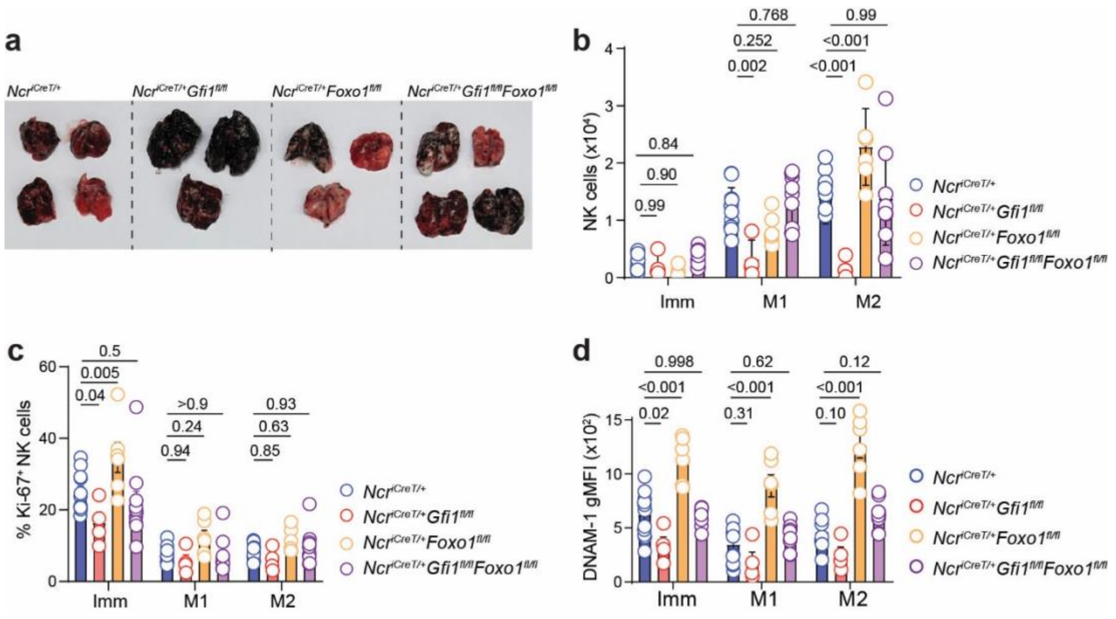


**Supplemental Figure 11. Combined loss of GFI1 and FOXO-1 restores NK cell tumour control. a**, Infiltration of B16-F10 tumour cells in the lungs of *$Ncr^{iCreT/+}$*, *$Ncr^{iCreT/+}Gfi1^{fl/fl}$*, *$Ncr^{iCreT/+}Foxo1^{fl/fl}$* and *$Ncr^{iCreT/+}Gfi1^{fl/fl}Foxo1^{fl/fl}$* mice at 14 days after tumour inoculation. Representative images show tumour metastases. Data representative of two independent experiments (*n*= 6, 9 or 11 biologically independent mice/ genotype)/ independent experiment. **b**, Total number of NK cell subsets isolated from lungs 14 days after B16-F10 tumour cell inoculation intravenously as in **a**. **c**, Frequency of Ki-67$^{+}$ NK cells in lungs as in **a**. **c**, DNAM-1 expression quantification in NK cell subsets in lungs as in **a**. **b**-**c**, Data pooled from two independent experiments shown as mean ± s.e.m. (*n*= 4, 6, 9 or 11 biologically independent mice/ genotype/ independent experiment). Statistical significance was calculated using a two-way ANOVA test with Dunnet's post-test (**b-d**).

## References


1. Narni-Mancinelli, E. *et al.* Fate mapping analysis of lymphoid cells expressing the NKp46 cell surface receptor. *PNAS* **108**, 18324-18329 (2011).
2. Thambyrajah, R. *et al.* GFI1 proteins orchestrate the emergence of haematopoietic stem cells through recruitment of LSD1. *Nat Cell Biol* **18**, 21-32 (2016).
3. Zhu, J., Jankovic, D., Grinberg, A., Guo, L. & Paul, W.E. Gfi-1 plays an important role in IL-2-mediated Th2 cell expansion. *Proceedings of the National Academy of Sciences of the United States of America* **103**, 18214-18219 (2006).
4. Kara, E.E. *et al.* CCR2 defines in vivo development and homing of IL-23-driven GM-CSF-producing Th17 cells. *Nat Commun* **6**, 8644 (2015).
