# Supplementary material for: The GFI1–FOXO1 axis regulates NK cell maturation and function
Source: Nat Commun. 2026 Apr 22;17:5593. doi: 10.1038/s41467-026-72022-6 (PMC13315798; doi:10.1038/s41467-026-72022-6)
Supplement: Supplementary file 2 — Description of Additional Supplementary Files [file 41467_2026_72022_MOESM2_ESM.pdf]

Description of Additional Supplementary Files

Supplemental Data 1. Pseudobulk RNA-seq analysis of total, cluster 1 (differentiated) and cluster 6 (immature) $Ncr^{iCreT/+}$ and $Ncr^{iCreT/+}Gfi1^{fl/fl}$ NK cells isolated from naïve mice. DEGs (p<0.05) for $Ncr^{iCreT/+}Gfi1^{fl/fl}$ *vs* $Ncr^{iCreT/+}$NK cells. Significance was determined by Wilcoxon Rank Sum test followed by Benjamini-Hochberg FDR testing.

Supplemental Data 2. Bulk RNA-seq of NK cells isolated from spleen of $Ncr^{iCreT/+}$ and $Ncr^{iCreT/+}Gfi1^{fl/fl}$ naïve mice. DEGs (p<0.05) for $Ncr^{iCreT/+}Gfi1^{fl/fl}$ *vs* $Ncr^{iCreT/+}$NK cells. Significance was determined by Wald's test followed by Benjamini-Hochberg FDR testing.

Supplemental Data 3. Pseudobulk ATAC-seq analysis of total, cluster 1 (differentiated) and cluster 6 (immature) $Ncr^{iCreT/+}$ and $Ncr^{iCreT/+}Gfi1^{fl/fl}$ NK cells isolated from naïve mice. DARs (p<0.05) for $Ncr^{iCreT/+}Gfi1^{fl/fl}$ vs $Ncr^{iCreT/+}$NK cells. Significance was determined by Wilcoxon Rank Sum test followed by Benjamini-Hochberg FDR testing.

Supplemental Data 4. Cluster-wise NK cell frequencies per genotype within each cluster across integrated dataset for $Ncr^{iCreT/+}$, $Ncr^{iCreT/+}Gfi1^{fl/fl}$, $Ncr^{iCreT/+}Foxo1^{fl/fl}$ and $Ncr^{iCreT/+}Gfi1^{fl/fl}Foxo1^{fl/fl}$ NK cells.

Supplemental Data 5. Cluster-specific marker genes identified by FindAllMarkers analysis for $Ncr^{iCreT/+}$, $Ncr^{iCreT/+}Gfi1^{fl/fl}$, $Ncr^{iCreT/+}Foxo1^{fl/fl}$ and $Ncr^{iCreT/+}Gfi1^{fl/fl}Foxo1^{fl/fl}$ NK cells after integration of four datasets. Significance was determined by Wilcoxon Rank Sum test followed by Benjamini–Hochberg FDR testing.

Supplemental Data 6. Pseudobulk RNA-seq analysis of $Ncr^{iCreT/+}$, $Ncr^{iCreT/+}Gfi1^{fl/fl}$, $Ncr^{iCreT/+}Foxo1^{fl/fl}$ and $Ncr^{iCreT/+}Gfi1^{fl/fl}Foxo1^{fl/fl}$ NK cells isolated from naïve mice. DEGs (p<0.05) for $Ncr^{iCreT/+}Gfi1^{fl/fl}Foxo1^{fl/fl}$ *vs* $Ncr^{iCreT/+}Gfi1^{fl/fl}$, $Ncr^{iCreT/+}Gfi1^{fl/fl}Foxo1^{fl/fl}$ *vs* $Ncr^{iCreT/+}$ and $Ncr^{iCreT/+}Foxo1^{fl/fl}$ *vs* $Ncr^{iCreT/+}$NK cells is shown. Significance was determined by Wilcoxon Rank Sum test followed by Benjamini-Hochberg FDR testing.

Supplemental Data 7. List of mouse strains and genotyping.

Supplemental Data 8. List of anti-mouse antibodies.

Supplemental Data 9. Reagent list.
