# Supplementary material for: The GFI1–FOXO1 axis regulates NK cell maturation and function
Source: Nat Commun. 2026 Apr 22;17:5593. doi: 10.1038/s41467-026-72022-6 (PMC13315798; doi:10.1038/s41467-026-72022-6)
Supplement: Supplementary file 12 — Reporting Summary [file 41467_2026_72022_MOESM12_ESM.pdf]

nature portfolio

Corresponding author(s): Gabrielle T. Belz

Last updated by author(s): Mar 21, 2026

Reporting Summary

Nature Portfolio wishes to improve the reproducibility of the work that we publish. This form provides structure for consistency and transparency in reporting. For further information on Nature Portfolio policies, see our Editorial Policies and the Editorial Policy Checklist.

Statistics

For all statistical analyses, confirm that the following items are present in the figure legend, table legend, main text, or Methods section.

n/a	Confirmed	
[ ]	[x]	The exact sample size (*n*) for each experimental group/condition, given as a discrete number and unit of measurement
[ ]	[x]	A statement on whether measurements were taken from distinct samples or whether the same sample was measured repeatedly
[ ]	[x]	The statistical test(s) used AND whether they are one- or two-sided<br>*Only common tests should be described solely by name; describe more complex techniques in the Methods section.*
[x]	[ ]	A description of all covariates tested
[ ]	[x]	A description of any assumptions or corrections, such as tests of normality and adjustment for multiple comparisons
[ ]	[x]	A full description of the statistical parameters including central tendency (e.g. means) or other basic estimates (e.g. regression coefficient) AND variation (e.g. standard deviation) or associated estimates of uncertainty (e.g. confidence intervals)
[ ]	[x]	For null hypothesis testing, the test statistic (e.g. *F*, *t*, *r*) with confidence intervals, effect sizes, degrees of freedom and *P* value noted<br>*Give P values as exact values whenever suitable.*
[x]	[ ]	For Bayesian analysis, information on the choice of priors and Markov chain Monte Carlo settings
[x]	[ ]	For hierarchical and complex designs, identification of the appropriate level for tests and full reporting of outcomes
[ ]	[x]	Estimates of effect sizes (e.g. Cohen's *d*, Pearson's *r*), indicating how they were calculated

Our web collection on statistics for biologists contains articles on many of the points above.

Software and code

Policy information about availability of computer code

Data collection	Flow cytometry analysis was performed on a Cytek Aurora (Cytek Biosciences) or LSRFortessa X-20 (BD Biosciences). Bulk RNA-seq and 10X single-cell multiome-seq libraries were sequenced using NovaSeq S1 PE100 flow cell (Illumina) or NovaSeq SP 100 flow cell (Illumina).
Data analysis	All flow cytometry data were analysed using FlowJo analysis software v.10.10 (BD Biosciences). Statistical analysis was performed using Prism v10.0 software (GraphPad Software).<br>For bulk RNA-seq, RNA-seq read quality was assessed and low quality reads trimmed with fastp v0.22.0. Reads were mapped to the mouse genome (mm10) using STAR v2.7.10 and quantified with featureCounts v2.0.1. Read counts were normalized and differential gene expression quantified with DESeq2 v1.4.0. A log-fold change larger than one and a false discovery rate cut-off of 5% was used to select significantly over- and under-represented genes. Geneset enrichment analysis was performed using clusterProfiler v4.8.3. Volcano plots and heatmaps were plotted using EnhancedVolcano v1.18.0 and pheatmap v1.0.12 packages, respectively.<br>Single-cell multiome sequencing data was analysed using CellRanger-arc, Seurat v5.0.3 and Signac v1.12.0. SCENIC+ v1.0a1 was used to identify gene regulatory networks. Topic modelling, dimensionality reduction, dropout imputation and differential accessibility region inference were performed using pycisTopic v2.0 with default parameters. Serial latent dirichlet allocation model with collapsed Gibbs sampler (500 iterations) was used for topic modelling. Topics ranged from 2 to 500, with the final model comprising 200 topics. PycisTarget v1.0 was used with default settings to incorporate cisTarget and differential enrichment of motifs using bulk consensus peaks motif database. The SCENIC+ was run with default parameters and http://ensembl.org/biomart/ was used as the biomaRt host. eRegulon results were filtered based on both the correlation between gene-based regulon area under the curve (AUC) and region-based regulon AUC with the cut off AUC > 0.7. Gene regulatory networks identified by SCENIC+ analysis were plotted using Cytoscape v3.10.0.

For manuscripts utilizing custom algorithms or software that are central to the research but not yet described in published literature, software must be made available to editors and reviewers. We strongly encourage code deposition in a community repository (e.g. GitHub). See the Nature Portfolio guidelines for submitting code & software for further information.

Data

Policy information about availability of data

All manuscripts must include a data availability statement. This statement should provide the following information, where applicable:

- Accession codes, unique identifiers, or web links for publicly available datasets
- A description of any restrictions on data availability
- For clinical datasets or third party data, please ensure that the statement adheres to our policy

Raw data files for the bulk RNA sequencing have been deposited in the NCBI Gene Expression Omnibus (GEO) under accession number GSE278867. Single-cell multiomic-seq data can be assessed from NCBI GEO accession number GSE313719.

Research involving human participants, their data, or biological material

Policy information about studies with human participants or human data. See also policy information about sex, gender (identity/presentation), and sexual orientation and race, ethnicity and racism.

Reporting on sex and gender	N/A
Reporting on race, ethnicity, or other socially relevant groupings	N/A
Population characteristics	N/A
Recruitment	N/A
Ethics oversight	N/A

Note that full information on the approval of the study protocol must also be provided in the manuscript.

Field-specific reporting

Please select the one below that is the best fit for your research. If you are not sure, read the appropriate sections before making your selection.

☒ Life sciences ☐ Behavioural & social sciences ☐ Ecological, evolutionary & environmental sciences

For a reference copy of the document with all sections, see nature.com/documents/nr-reporting-summary-flat.pdf

Life sciences study design

All studies must disclose on these points even when the disclosure is negative.

Sample size	Animal models used in this study are well established and based on prior research conducted in our laboratories to use sufficient numbers of mice or cells in each group. For each experiment 3-5 animals were used and experiments were repeated
Data exclusions	No data points were excluded.
Replication	All experiments were performed at least twice. The presented data were successfully replicated.
Randomization	Age- and sex-matched mice were allocated to groups based on the experimental treatment (no randomisation).
Blinding	Blinding was not performed in this study as data analysis in strictly quantitative (and not subjective) and data acquisition occurred based on fixed protocols. Thus, introduction of investigator bias is unlikely.

Reporting for specific materials, systems and methods

We require information from authors about some types of materials, experimental systems and methods used in many studies. Here, indicate whether each material, system or method listed is relevant to your study. If you are not sure if a list item applies to your research, read the appropriate section before selecting a response.

Materials & experimental systems

n/a	Involved in the study
☐	☒ Antibodies
☐	☒ Eukaryotic cell lines
☒	☐ Palaeontology and archaeology
☐	☒ Animals and other organisms
☒	☐ Clinical data
☒	☐ Dual use research of concern
☒	☐ Plants

Methods

n/a	Involved in the study
☒	☐ ChIP-seq
☐	☒ Flow cytometry
☒	☐ MRI-based neuroimaging

Antibodies

Antibodies used

a4b7, clone DATK32, APC Thermo Fisher Scientific Cat# 17-5887-80 1/200
BCL2, clone 10C4, PE/Cy7 Thermo Fisher Scientific Cat# 25-6992-42 1/100
CD11b, clone M1/70, FITC BD Biosciences Cat# 553310 1/400
CD11b, clone M1/70, BV510 BD Biosciences Cat# 562950 1/200
CD11b, clone M1/70, eFluro450 BD Biosciences Cat# 560455 1/200
CD11b, clone M1/70, BUV737 BD Biosciences Cat# 612801 1/200
CD122, clone TM-b1, PerCP-eFluor 710 Thermo Fisher Scientific Cat# 46-1222-82 1/100
CD122, clone TM-b1, BV650 BD Biosciences Cat# 740475 1/100
CD117 (c-Kit) clone 2B8, PerCP-eFluor 710, Thermo Fisher Scientific Cat# 46-1171-80 1/100
CD16/32, clone 2.4G2 BD Biosciences Cat# 553142 1/200
CD19, clone 1D3, BUV395 BD Biosciences Cat# 563557 1/200
CD19, clone 1D3, BV605 BD Biosciences Cat# 563148 1/200
CD127 (IL-7Rα), clone A7R34, BV421 BioLegend Cat# 135024 1/200
CD127 (IL-7Rα), clone SB/199, PE/Cy7 BD Biosciences Cat# 560733 1/200
CD226 (DNAM1), clone TX42.1, BV421 BioLegend Cat# 133615 1/100
CD253 (TRAIL), clone N2B2, PE Thermo Fisher Scientific Cat# 12-5951-82 1/100
CD27, clone LG.3A10, BV510 BioLegend Cat# 124229 1/100
CD3e, clone 145-2C11, BUV395 BD Biosciences Cat# 563565 1/200
CD3e, clone 145-2C11, BUV615 Thermo Fisher Scientific Cat# 366-0031-82 1/200
CD335 (NKp46) clone 29A1.4, PE, Thermo Fisher Scientific Cat# 12-3351-82 1/200
CD335 (NKp46) clone 29A1.4, PE/Cy7 Thermo Fisher Scientific Cat# 25-3351-82 1/200
CD335 (NKp46) clone 29A1.4, APC Thermo Fisher Scientific Cat# 17-3351-82 1/200
CD4, clone GK1.5, BUV496 BD Biosciences Cat# 564667 1/200
CD4, clone RM4-5, AF700 Thermo Fisher Scientific Cat# 11-0041-85 1/200
CD44, clone IM7, BUV496 BD Biosciences Cat# 741057 1/500
CD45, clone 30-F11, BV786 BD Biosciences Cat# 748370 1/1000
CD45.1, clone A20, FITC BD Biosciences Cat# 553775 1/200
CD45.1, clone A20, APC/eFluor 780 Thermo Fisher Scientific Cat# 47-0453-82 1/200
CD45.2, clone 104, eFluor 450 Thermo Fisher Scientific Cat# 48-0454-82 1/200
CD45.2, clone 104, Pacific Blue BioLegend Cat# 109832 1/200
CD49a, clone Ha31/8, BUV496 BD Biosciences Cat# 741111 1/200
CD49a, clone Ha31/8, AF647 BD Biosciences Cat# 562113 1/200
CD49b, clone HMa2, BUV661 BD Biosciences Cat# 741523 1/200
CD49b, clone DX5, BV421 BD Biosciences Cat# 563063 1/200
CD49b, clone DX5, PE/eF610 Thermo Fisher Scientific Cat# 61-5971-82 1/200
CD62L, clone MEL-14, Pacific Blue Thermo Fisher Scientific Cat# PA5-60507 1/1000
CD62L, clone MEL-14, BV786 BD Biosciences Cat# 564109 1/1000
CD8a, clone 53-6.7, PerCP/Cy5.5 BD Biosciences Cat# 551162 1/100
CD8a, clone 53-6.7, BUV737 BD Biosciences Cat# 564297 1/200
CX3CR1, clone SA011F11, BV605 BioLegend Cat# 149027 1/500
CXCR3, clone CXCR3-173, BUV737 BD Biosciences Cat# 741895 1/200
EOMES, clone Dan11mag, PE/Cy5 BD Biosciences Cat# 15-4875-82 1/100
EOMES, clone Dan11mag, PE-eFluor 610 BD Biosciences Cat# 61-4875-82 1/100
FOXO1, clone C29H4, PE Cell Signaling Technology Cat# 14262 1/100
Granzyme B, clone NGZB, PE/Cy7 Thermo Fisher Scientific Cat# 25-8898-82 1/100
IFNg, clone XMG1.2, PE BioLegend Cat# 505808 1/100
Ki-67, clone SolA15, AF488 Thermo Fisher Scientific Cat# 53-5698-82 1/200
Ki-67, clone B56, BUV395 BD Biosciences Cat# 564071 1/100
KLRG1, clone 2F1, BV711 BD Biosciences Cat# 564014 1/200
KLRG1, clone 2F1, PE/Cy7 Thermo Fisher Scientific Cat# 25-5893-82 1/500
NK1.1, clone PK136, BV650 BD Biosciences Cat# 564143 1/500
NK1.1, clone PK136, APC Thermo Fisher Scientific Cat# 17-5941-82 1/500

	Perforin, clone eBioOMAK-D, APC Thermo Fisher Scientific Cat# 17-9392-80 1/100<br>PD1, clone J43, PE/CF594 BD Biosciences Cat# 562523 1/100<br>RORgt, clone Q31-378, BV421 BD Biosciences Cat# 562894 1/300<br>Sca-1, clone D7, V500 BD Biosciences Cat# 561228 1/500<br>Sca-1, clone D7 SB645 Thermo Fisher Scientific Cat# 64-5981-82 1/100<br>T-BET, clone eBio4B10, PE/Cy5 Thermo Fisher Scientific Cat# 15-5825-82 1/100<br>T-BET, clone eBio4B10, PE/Cy7 Thermo Fisher Scientific Cat# 25-5825-82 1/100<br>TCF1, clone S33-966, AF488 BD Biosciences Cat# 567018 1/100<br>TCF1, clone S33-966, PE BD Biosciences Cat# 564217 1/200<br>TCR β clone H57-597, APC-eFluor 780 Thermo Fisher Scientific Cat# 47-5961-82 1/200<br>TCR β clone H57-597, PE/Cy7 BioLegend Cat# 109222 1/500<br>TNF, clone MP6-XT22, BV421 BD Biosciences Cat# 563387 1/100<br>TNF, clone MP6-XT22, APC Thermo Fisher Scientific Cat# 17-7321-82 1/100<br>TIM3, clone 5D12, PE BD Biosciences Cat# 566346 1/200<br>TOX, clone TXRX10, eFluor 660 Thermo Fisher Scientific Cat# 50-6502-82 1/100
Validation	All antibodies were titrated and validated using appropriate controls.

Eukaryotic cell lines

Policy information about cell lines and Sex and Gender in Research

Cell line source(s)	B16-F10 (CRL-6475) and M2-10B4 (CRL-1972) were originally obtained from ATCC.
Authentication	Not authenticated
Mycoplasma contamination	None detected.
Commonly misidentified lines<br>(See ICLAC register)	No commonly misidentified lines were used in this study.

Animals and other research organisms

Policy information about studies involving animals; ARRIVE guidelines recommended for reporting animal research, and Sex and Gender in Research

Laboratory animals	C57BL/6 (CD45.1+ or CD45.2+), Gfi1-tdTomato, Gfi1fl/fl, Foxo1tm1Rdp, Eomestm1Gtbz (Eomesfl/fl) and NcriCre were used. Both, male and female mice were used at 6-16 weeks old age. All mice were bred and maintained under specific pathogen-free conditions at the animal facility of The University of Queensland. Mice were housed under a 12-hours light/12-hours dark cycle at 22°C ± 2°C and 55% ± 15% humidity.
Wild animals	No wild animals were used in this project.
Reporting on sex	Both, male and female mice were used.
Field-collected samples	None
Ethics oversight	The University of Queensland ethics committee.

Note that full information on the approval of the study protocol must also be provided in the manuscript.

Plants

Seed stocks	*Report on the source of all seed stocks or other plant material used. If applicable, state the seed stock centre and catalogue number. If plant specimens were collected from the field, describe the collection location, date and sampling procedures.*
Novel plant genotypes	*Describe the methods by which all novel plant genotypes were produced. This includes those generated by transgenic approaches, gene editing, chemical/radiation-based mutagenesis and hybridization. For transgenic lines, describe the transformation method, the number of independent lines analyzed and the generation upon which experiments were performed. For gene-edited lines, describe the editor used, the endogenous sequence targeted for editing, the targeting guide RNA sequence (if applicable) and how the editor was applied.*
Authentication	*Describe any authentication procedures for each seed stock used or novel genotype generated. Describe any experiments used to assess the effect of a mutation and, where applicable, how potential secondary effects (e.g. second site T-DNA insertions, mosiacism, off-target gene editing) were examined.*

Flow Cytometry

Plots

Confirm that:

- [x] The axis labels state the marker and fluorochrome used (e.g. CD4-FITC).
- [x] The axis scales are clearly visible. Include numbers along axes only for bottom left plot of group (a 'group' is an analysis of identical markers).
- [x] All plots are contour plots with outliers or pseudocolor plots.
- [x] A numerical value for number of cells or percentage (with statistics) is provided.

Methodology

Sample preparation	Single cell suspensions were generated by forcing tissues through 70m cell strainers, and red blood cells (RBC) were removed using hypotonic lysis using ACK buffer. Cell suspensions were blocked with PBS containing 5ug/ml anti-CD16/CD32 and stained (30 min on ice) with fluorophore-conjugated antibodies or reagents in FACS buffer. For intracellular staining, surface-labelled cells were fixed using eBioscience Foxp3/Transcription Factor Staining Buffer (Thermo Fisher) then stained for intracellular cytokines or transcription factors. Live cells were identified by exclusion staining with a fixable viability dye (BD Biosciences or BioLegend) or 7-AAD (BD Biosciences).
Instrument	Flow cytometry analysis was performed on a Cytek Aurora (Cytek Biosciences) or LSRFortessa X-20 (BD Biosciences). Cell Sorting was performed using BD FACS aria II (BD Biosciences).
Software	All flow cytometry data were analysed using FlowJo analysis software v.10.10 (BD Biosciences). Statistical analysis was performed using Prism v10.0 software (GraphPad Software).
Cell population abundance	Cell populations analysed ranged from 50-1000000 cells/mouse.
Gating strategy	1. FSC-A/SSC-A were used to select lymphocyte populations.<br>2. FSC-A/FSC-H were used to select singlets.<br>3. FSC-A/viability were used to identify live cells.<br>4. TCRb/CD19 were used to gate CD19-TCRb- cells.<br>5. NK1.1/Nkp46 were used to gate NKp46+ cells.<br>6. CD49a/CD49b were used to gate NK cells.

- [x] Tick this box to confirm that a figure exemplifying the gating strategy is provided in the Supplementary Information.
